# Non-canonical Helitrons in *Fusarium oxysporum*

**DOI:** 10.1186/s13100-016-0083-7

**Published:** 2016-12-09

**Authors:** Biju Vadakkemukadiyil Chellapan, Peter van Dam, Martijn Rep, Ben J. C. Cornelissen, Like Fokkens

**Affiliations:** 1 Department of Computational Biology and Bioinformatics, University of Kerala, Karyavattom Campus, Karyavattom PO, Trivandrum, Kerala India; 2 Molecular Plant Pathology, Swammerdam Institute for Life Sciences, Faculty of Science, University of Amsterdam, P.O. Box 94215, 1090 Amsterdam, GE The Netherlands

**Keywords:** Helitrons, Transposon, Rolling circle, Terminal inverted repeats, Helitron2, Helentrons, *Fusarium oxysporum*

## Abstract

**Background:**

Helitrons are eukaryotic rolling circle transposable elements that can have a large impact on host genomes due to their copy-number and their ability to capture and copy genes and regulatory elements. They occur widely in plants and animals, and have thus far been relatively little investigated in fungi.

**Results:**

Here, we comprehensively survey Helitrons in several completely sequenced genomes representing the *F. oxysporum* species complex (FOSC). We thoroughly characterize 5 different Helitron subgroups and determine their impact on genome evolution and assembly in this species complex. FOSC Helitrons resemble members of the Helitron2 variant that includes Helentrons and DINEs. The fact that some Helitrons appeared to be still active in FOSC provided the opportunity to determine whether Helitrons occur as a circular intermediate in FOSC. We present experimental evidence suggesting that at least one Helitron subgroup occurs with joined ends, suggesting a circular intermediate. We extend our analyses to other Pezizomycotina and find that most fungal Helitrons we identified group phylogenetically with Helitron2 and probably have similar characteristics.

**Conclusions:**

FOSC genomes harbour non-canonical Helitrons that are characterized by asymmetric terminal inverted repeats, show hallmarks of recent activity and likely transpose via a circular intermediate. Bioinformatic analyses indicate that they are representative of a large reservoir of fungal Helitrons that thus far has not been characterized.

**Electronic supplementary material:**

The online version of this article (doi:10.1186/s13100-016-0083-7) contains supplementary material, which is available to authorized users.

## Background

Transposable elements (TEs) are stretches of DNA that are able to copy or move from one site to another in a genome. Autonomous TEs contain one or more sequences coding for proteins that are involved in transposition, combined with TE-specific DNA motifs such as terminal inverted repeats. These motifs are required for transposition. Non-autonomous elements possess the DNA motifs but do not encode a functional transposase. They profit from their autonomous counterparts and often greatly outnumber them.

Helitrons are a family of TEs that encode an Y2-transposase consisting of an N-terminal rolling circle replication initiator (Rep) domain and a C-terminal helicase (Hel) domain. They were first characterized in an *in silico* analysis of the genomes of *A. thaliana*, *O. sativa* and *C. elegans* [1], where they were found to have a 5’-TC and 3’-CTRR (where R stands for A or G) motif and a short hairpin at 10–12 nucleotides distance from the 3’ terminus. Recent reports indicate that Helitrons can be divided into two groups: Helitron1 and Helitron2 [2–4]. The motifs that were found upon first discovery of Helitrons are specific to the Helitron1. In contrast Helitron2 TEs are characterized by an asymmetric terminal inverted repeat (ATIR) and a hairpin at both termini. Helentrons cluster phylogenetically with Helitron2 proteins and possess similar termini, but, in addition to the Rep and Hel domains, they possess an endonuclease domain that they obtained through insertion of a retrotransposon [2–6]. DINEs, also known as HINEs, the most abundant TE in Drosophila, are non-autonomous elements derived from Helentrons [3] (see [7] for a recent review).

Recent in-depth analyses of a mobile pathogenicity chromosome of the ascomycete *Fusarium oxysporum* f. sp. *lycopersici* strain Fol4287 revealed 9 nearly identical genes encoding proteins with a Rep-Hel domain architecture [8]. The *Fusarium oxysporum* species complex (FOSC) consists of clonal lines of *Fusarium oxysporum*, a filamentous fungus that colonizes plant roots and occasionally enters the plant’s roots and vascular system, causing wilting or root-rot disease symptoms. Individual pathogenic strains are usually pathogenic to only a small number of related host plants, but the species complex as a whole is a versatile pathogen with great economic impact [9]. *Fusarium oxysporum* represents an extreme case of a two-speed genome: its chromosomes can be classified as either ‘core’ or ‘lineage specific’ (LS), where core chromosomes are largely syntenic with chromosomes of other *Fusarium* species, while LS chromosomes are largely absent in other *Fusarium* species [10–12]*.* The LS chromosomes are enriched in TEs and in genes involved in pathogenicity. Genomes of 12 strains of this species complex have been sequenced, assembled and annotated [13], providing an excellent dataset for a thorough study of Helitrons in an ascomycete.

The genomic impact of Helitrons, in terms of copy number as well as in terms of whether Helitrons inserted in or near genes, varies strongly between different species (see [7] for a recent review). This depends on transposition efficiency and effectiveness of TE silencing, but also on whether we are observing a host genome that experienced a recent Helitron outbreak versus the remnants of past activity. In the latter case we expect for example that Helitron copies that adversely affect coding or regulatory regions or gene regulation have been removed from a population through purifying selection. A factor that is often overlooked is the completeness of genome assembly. Within our FOSC dataset, the genomes are assembled up to different levels of completeness, which allows us to assess the impact of incomplete genome assembly on copy number estimates.

A recent study using a reconstructed ancestral bat Helitron1 sequence provided important insights into the mechanisms underlying transposition and gene capture in canonical Helitrons [14]. First of all, the authors could demonstrate that Helitrons transpose as single stranded DNA. This is congruent with the fact that Helitrons do not cause target site duplications that are associated with double stranded, staggered breaks. Recent biochemical studies show that they transpose via copy-paste rather than cut-and-paste, which explains their high copy number [14]. Helitrons can capture (parts of) genes and thus contribute to the emergence of new genes through combining of different coding and non-coding sequence that have been sequentially captured [4, 6, 7, 14–22]. Grabundzija and others confirmed the ‘end-bypass’ model of gene capture in Helitrons, in which the transposase skips the 3’ terminus and thus includes 3’ flanking DNA sequence in the excised Helitron. Finally, Grabundzija and others demonstrated that canonical Helitrons occur as a circular intermediate [14], as has been observed previously for the Insertion Sequence IS91 in *Escherichia coli* [23]. Transposition via a circular intermediate can also explain the presence of multiple tandem insertions of truncated Helitrons that have recently been found in plant centromeres [24]. This indicates that the processes of excision and insertion are decoupled in Helitrons. We extensively survey footprints of past Helitron activity, focussing on putative Helitron self-insertions, to shed light on the transposition process in FOSC Helitrons.

Helitrons are found in a wide range of eukaryotes, including plants, animals, fungi and oomycetes, but have predominantly been described in plants and animals [1, 4–6, 15, 18, 21, 25–37]. We ask whether FOSC Helitrons are relatively unique or whether they represent a larger and relatively unknown reservoir of Pezizomycotina Helitrons. Finally, we study conservation of terminal sequences and ask how the Helitrons we uncovered are related to the two known Helitron families.

## Results

### FOSC Helitrons divide into two groups and 5 subgroups

**Fig. 1:**
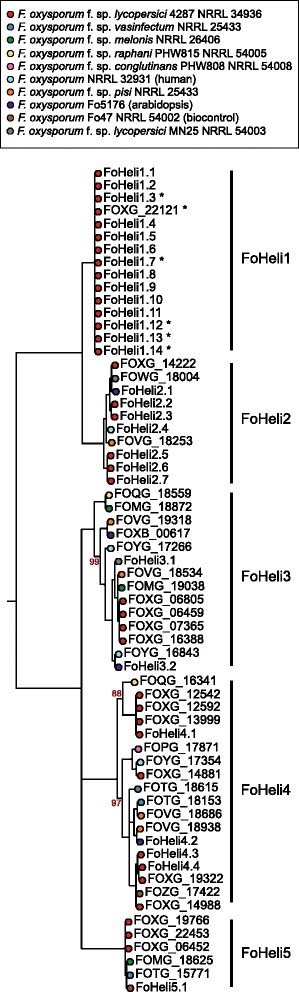
FOSC Helitrons divide into two groups and 5 subgroups. The phylogeny inferred from a multiple sequence alignment of the (predicted) protein sequences of FOSC Helitrons shows that they are separated into two major groups that can be further subdivided into 5 subgroups in total. These subgroups are designated FoHeli1 – FoHeli5. All except three branches have 100% bootstrap support, the bootstrap support (based on 100 replicates) of those three is indicated in red adjacent to the respective branch. Nodes are coloured according to the fungal strain the Helitron was found in. FoHeli1 is distinct from the other 4 subgroups because the protein sequences are nearly identical, because this subgroup is only found in the most completely assembled genome, that of Fol4287, and because this subgroups is also found on core chromosomes of Fol4287. Copies on core chromosomes are indicated with an *. In the genomes of strains *F. oxysporum* f. sp. *radicis-lycopersici* CL57 and *F. oxysporum* f.sp. *cubense* II5 no genes encoding proteins with a Rep-Hel domain architecture were detected. This is most likely due to deficiencies in genome assembly and gene annotation as we do find partial Helitron copies in these genomes, albeit in small numbers (Additional file 5: Table S5)

Most software designed to identify Helitrons are based on the DNA motifs of the Helitron1 variant and will overlook instances of Helitron2 because these have different termini [5, 18, 20, 38–40]. Moreover, DNA sequence similarity can be hard to recognize over long evolutionary distances and very few ascomycete Helitron sequences were available at the start of our studies. Therefore we selected 35 FOSC proteins with a Rep—Hel domain architecture and used those to search the FOSC genomes for additional, unannotated genes that encode putative Helitron proteins. We found in total 63 proteins in 10 different strains that encode proteins with the typical Helitron domain architecture and named them FoHelis (Fig. 1). Conserved motifs within the Rep as well as the Hel domain are present in most FOSC Helitrons, suggesting that these proteins are functional (Additional file 1: Figure S1 and Figure S2) [41–43]. Like other Helitrons, the putative Helitron proteins we predicted in FOSC have an N-terminal zinc finger-like motif (Additional file 1: Figure S3) [5].

To distinguish different subgroups, we inferred a phylogenetic tree for these 63 protein sequences. We found that they divide into two major groups and five subgroups: FoHeli1 and FoHeli2 in group I, and FoHeli3—FoHeli5 in group II (Fig. 1). FoHeli1 is the subgroup identified earlier [8] and differs from the other subgroups in several respects: (i) they’re found only in the genome of *F. oxysporum* f. sp. *lycopersici* Fol4287 (hereafter referred to as Fol4287) among the 12 strains, (ii) they’re found on many different chromosomes, including core chromosomes (Fig. 1, Additional file 2: Table S1) and (iii) there is very little sequence diversity in this subgroup.

### FoHeli termini are non-canonical and resemble those of the Helitron2 variant

**Fig. 2:**
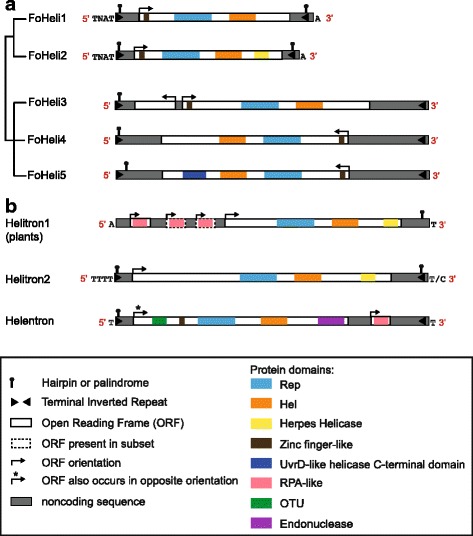
Terminal features and coding capacity for FOSC and other Helitrons. **a** FoHeli termini are characterized by hairpins and inverted repeats, where the 3’ inverted repeats is ~20–40 bp upstream from the terminal sequence. Within each of the two groups, termini are very similar. FoHeli1 and FoHeli2 have two hairpins, one at each terminus, a 12 bp long inverted repeat, start with ‘TCAG’ and end with ‘ATTTT’. Similar to canonical Helitrons, the 3’ inverted repeat and hairpin are located at ~30-40 bp from the 3’ terminus. In the other group, all FoHelis start with ‘TGCCT’ and end with ‘CTCCTGT’. At the 5’ end, they have a hairpin but they lack a hairpin at the 3’ end. The ORFs in FoHeli4 and FoHeli5 have an opposite orientation when compared to FoHeli3. FoHeli1 and FoHeli2 insert between ‘TNAT’ and ‘T’, for the other group we could not establish an insertion preference. **b** When we compare these structural features to those of known Helitrons, we find that FOSC Helitrons resemble Helitron2 transposons. Structural features of Helitron1 (canonical Helitrons), Helitron2 and Helentrons were compiled from [2, 7] and RepBase [51]. Helitron1/canonical Helitrons insert between ‘A’ and ‘T’, Helitron2 between ‘TTTT’ and ‘T’ or ‘C’ and Helentrons in a ‘TT’ dinucleotide. See Additional file 1: Figures S13 and S14 for more detail on Helitron domain composition

Using multiple sequence alignments for sets of similar sequences within each subgroup, we identified termini for 48 out of 63 Helitrons, despite the fact that many Helitrons reside on the borders of contigs or supercontigs. More importantly, we found termini for members of each subgroup (Additional file 2: Table S1, Fig. 2a). Interestingly, all FoHeli termini we have identified include asymmetric terminal inverted repeats (ATIRs), like members of the Helitron2 variant. In addition, FoHeli1 and FoHeli2 have hairpins at both termini, as is also observed in some Helitron2 TEs.

Within each of the two major groups, the sequences of the termini are very similar: subgroups FoHeli1 and FoHeli2 have “**TCAGCC** **GAA** **GGCTG** AC” and “T[c/a]**AGTCC** **GAA** **GGA** CTT”, respectively, at the 5’ end, where underlined nucleotides indicate the stem of a hairpin. Nucleotides in bold are present as an inverted repeat, that is itself also part of a hairpin, at the 3’ end of the element, 38 (FoHeli2) to 51 (FoHeli1) bp upstream from their 3’ terminus ‘ATATTTT’. The distance between the termini (i.e. the length of the full Helitron transposable element) is quite short: ~6 kb for FoHeli1 and ~5 kb for FoHeli2 (Additional file 2: Table S1). In the other major group, subgroups FoHeli3-FoHeli5 have “TGCCT” and a degenerate hairpin at the 5’ end, and “CTCCTGT” at the 3’ end, combined with an inverted repeat of between 13–16 bp. The distance between termini is much larger in this group, ranging from ~9 to ~11 kb (Additional file 2: Table S1).

**Fig. 3:**
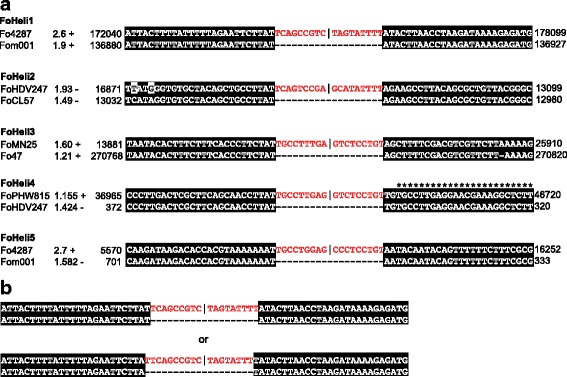
**a** Alignment of insertion sites confirms FoHeli termini. For each subgroup, we reconstruct pre-insertion sites by concatenating FoHeli flanking sequences and search for these pre-insertion sequences in our set of FOSC genomes. Alignment of FoHeli flanking sequenes with these pre-insertion sites showed that the termini we had inferred before are correct. Fom001 – *F. oxysporum* f.sp. *melonis* 26406, FoMN25 - *F. oxysporum* f.sp. *lycopersici* MN25, FoHDV247 - *F. oxysporum* f.sp. *pisi* HDV247, FoCL57 - *F. oxysporum* f.sp. *radicis-lycopersici* CL57, FoPHW815 - *F. oxysporum* f.sp. *raphani* PHW815, FoPHW808 - *Fusarium oxysporum* f.sp. *conglutinans* PHW808. In this example a FoHeli4 is inserted 2 bp from another FoHeli4 (indicated with * above the sequence). **b** Because FoHelis have a ‘T’ at the 5’ terminus and preferentially insert between ‘T’ and ‘A’, we can not be certain that e.g. FoHeli1s and FoHeli2s start with ‘TC’ like canonical Helitrons, or with ‘TTC’.

Alignment of reconstructed pre-insertion sites confirmed that the termini we found are correct (Fig. 3a). In contrast to what has been reported on Helentrons [3], we have not observed variations in the number of Ts at 3’ ends. Canonical Helitrons insert preferentially into an ‘AT’ dinucleotide. The preferred insertion site for FoHeli1 and FoHeli2 is between ‘TNAT’ and ‘A’, where ‘N’ denotes any nucleotide (Figs. 2a and 3a). Note that because FoHeli1 and FoHeli2 have a ‘T’ at the 5’ terminus and preferentially insert between ‘T’ and ‘A’, we can not be certain that FoHeli1s and FoHeli2s start with ‘TC’ like canonical Helitrons, or with ‘TTC’ (Fig. 3b). From here, we assume that FoHeli1 and FoHeli2 start with ‘TC’, like canonical Helitrons.

In subgroup FoHeli1, two copies are 100% identical from 5’ to 3’ terminus: FoHeli1.11 on chromosome 14 and FoHeli1.15 (FOXG_22121) on chromosome 8. Within this subgroup all copies are more than 99% identical to eachother, from terminus to terminus. This suggests that FoHeli1 has been active relatively recently and may still be active. The other subgroups do contain identical copies, but these lie in regions that are part of large segmental duplications in Fol4287 and are not the result of recent transposition (Additional file 1: Figure S4, [10]). Only FoHeli2 has two members for which both termini have been identified, that are on the same genome and not interrupted by contigbreaks. FoHeli2.8 (FOXG_14222) and FoHeli2.2 are 98.76% identical from the 5’ to the 3’ terminus. For the other subgroups the period of activity can not be compared based on sequence divergence.

Several FoHelis have multiple predicted Open Reading Frames (ORFs) but most ORFs overlap with the Helitron transposase and are probably the result of gene prediction errors (Fig. 2, Additional file 3: Table S2). FoHeli3 is the only subgroup with a predicted ORF that does not overlap with the gene encoding the transposase. This additional ORF is located upstream from the transposase gene and has an opposite orientation. It has no known domains and only occurs in Helitron TEs (Additional file 3: Table S2). Several plant Helitrons contain one or more genes encoding an RPA-like protein; we found no RPA-like genes in FoHelis. Interestingly, the transposase ORFs in FoHeli4 and FoHeli5 have an inverted orientation when compared to FoHeli3 in the same major group (Fig. 2). This phenomenon has been observed before in Helitron2-like elements: in a Helentron in the fish *Danio rerio*, a Helentron in the fruit fly *Drosophila ananassae* and in a Helitron2 in the green alga *Chlamydomonas reinhardtii* [2, 3].

### FOSC genomes contain non-autonomous FoHelis

In plant and animal genomes, the most abundant Helitrons are non-autonomous; they possess the structural terminal features that are needed for transposition, but do not encode a functional transposase. They are typically much shorter than autonomous Helitrons. The fact that we have terminus-to-terminus sequences for each subgroup allowed us to query the 12 FOSC genomes for non-autonomous elements. We found two types of non-autonomous elements in which (part of) the Helitron coding sequence was deleted. Interestingly, these non-autonomous elements all appear to have derived from FoHeli1, and we found them only in genomes in which we could not find a putative autonomous FoHeli1 copy. Moreover, their high sequence similarity and distinct termini suggest they have recently transposed.

The shortest element of the two, named FoHeliNA1, is 830 bp in size. We found this element in low copy number in the genomes of *F. oxysporum* f. sp. *raphani* PHW815, *F. oxysporum* f. sp. *vasinfectum, F. oxysporum* f. sp. *conglutinans* PHW808 and *F. oxysporum* Fo5176 (Additional file 4: Table S3). Its first 27 bp and last 166 bp are, respectively, ~92.5% identical to the 5’ and ~78.2% identical to the 3’ terminus of FoHeli1. The 637 bp between the termini are not similar to any of the Helitrons we had identified before (Additional file 1: Figure S5). The second type of non-autonomous element, named FoHeliNA2, is 1929 bp in size and was found in the genomes of *F. oxysporum* f.sp. *raphani* PHW815, *F. oxysporum* f.sp. *vasinfectum*, *F. oxysporum* NRRL 32931 and *F. oxysporum* f.sp. *pisi* HDV247, again in low copy number. Its first 1092 and last 837 bp are ~90% identical to FoHeli1 termini (Additional file 1: Figure S6). One copy of FoHeliNA2 has inserted into a putative autonomous FoHeli, namely FoHeli3.3 (FOQG_18559) in *Fusarium oxysporum* f.sp. *raphani* PHW815.

Increasing the maximum distance between matching termini allowed us to detect a few full-length Helitrons that were previously unrecognized, mostly because no or an incomplete ORF was predicted. Possibly, these Helitrons have pseudogenized, or the presence of assembly gaps in the coding sequence has hampered the correct prediction of the ORF. We also identified a few cases in which a hAT or a Hornet TE was inserted into a Helitron, truncating the ORF (Additional file 2: Table S1, Additional file 4: Table S3), but found no evidence that these ‘chimeric’ TEs have transposed (Additional file 1: Figure S4).

### FoHeli copy number is underestimated due to genome assembly being hampered by the presence of identical FoHeli copies

The presence of non-autonomous Helitrons in genomes that do not have an autonomous version suggests that we may have failed to identify the putative autonomous copies in these genomes. Most FOSC genome sequences are based on short reads generated by second-generation sequencing. The occurrence of multiple, highly similar copies of a long sequence, due to recent gene duplications or recent transposition of TEs, greatly impacts these assemblies. Single reads only cover a small section of the repeated sequence and for those reads that do not contain a portion of unique flanking sequence, it is impossible to infer to which copy they belong. Most assemblers introduce a contig break and assemble all reads that fall completely within the repeated sequence into a single contig with very high coverage [44, 45].

If incomplete genome assembly hampered the detection of Helitrons, we should find partial Helitron copies at the borders of contigs and supercontigs, and some contigs that consist entirely of a Helitron sequence. Indeed, when we query the 12 FOSC genomes with DNA sequences of full-length (terminus-to-terminus) elements, we find that for FoHeli1, FoHeli2 and FoHeli4, most partial copies are located near the edge of a (super)contig (Additional file 5: Table S5). Especially the presence of FoHeli1 and FoHeli2 copies seem to have impaired genome assembly: respectively 82% and 96% of partial copies are located near contig borders, or span entire contigs, compared to 32% to 68% percent of FoHeli3—FoHeli5. Notably, a large fraction of these partial copies are between 80 and 150 bp long, which is what is expected given the read length that was achieved on Illumina platforms at the time these genomes were sequenced.

Conversely, due to incomplete genome assembly, the copy number of Helitrons in FOSC is potentially severely underestimated. If we assume that every Helitron ‘start’ is actually an unrecognized complete (potentially non-autonomous) copy, counting multiple termini as one, we arrive at an upper-bound copy number estimate that is almost ten-fold higher than the number of Helitrons we identified in our initial search (Additional file 5: Table S5). In total we then predict 559 copies in the FOSC, where FoHeli1 and FoHeli2 are most abundant with 115 and 327 copies in all 12 strains, respectively. Notably, FoHeli2 is particularly abundant in strains that are able to infect Arabidopsis (*F. oxysporum* f. sp. *conglutinans* PHW808: 95, *F. oxysporum* Fo5176:147 and *F. oxysporum* f. sp. *raphani* PHW815: 54), whereas other subgroups are more evenly distributed among the different strains.

### Amplicons with the sequence of FoHeli1 with joined ends suggest presence of a circular intermediate

**Fig. 4:**
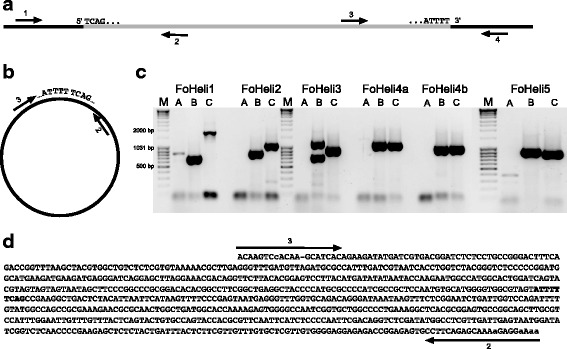
Putative circular Helitrons detected by PCR. **a** Schematic representation of FoHeli1 in the genome. The grey line represents FoHeli1 and the 5’ and 3’ terminal sequences are indicated above. The black thick lines represent the flanking genomic region. The arrows indicate the positions of the primers. For each subgroup, FoHeli1 to FoHeli5, we designed four specific PCR primers (Additional file 1: Table S4). Primer pairs 1 + 2 and 3 + 4 are specific to FoHeli 5’ and 3’ ends and their flanking sequences, respectively. Primers 2 + 3 anneal close to FoHeli ends and are directed outwards; these are expected to amplify a PCR product only from molecules that contain nearby or joined FoHeli ends. **b** Schematic representation of a FoHeli1 circle with joined ends (possible template for the amplification of a PCR product using primers 2 + 3) **c** PCR experiment showing amplification of PCR products using primer pair A (primers 2 + 3), B (primers 1 + 2) and C (primers 3 + 4) specific for FoHeli1 – FoHeli5. The template for the PCR reaction was genomic DNA isolated from Fol4287. We used two sets of primers for FoHeli4, because this subgroup is more divergent than the others. Note that there is ~400 bp PCR product of FoHeli5 using outward directed primers. However, the sequence of FoHeli5 with joined ends between these primers is 570 bp. Moreover, the sequence of this amplicon did not show any similarity to a FoHeli. Hence we concluded that this amplicon does not correspond to a FoHeli5 with joined ends. **d** Structure of FoHeli1 joined ends. The terminal sequences are shown in bold

A recent study demonstrated that canonical Helitrons transpose via a circular intermediate [14]. We tested for the presence of a FoHeli circle in Fol4287 by trying to amplify the junction sequence of FoHeli with joined ends by PCR, using primers that anneal close to termini of FoHeli and are directed outwards, and genomic DNA from Fol4287 as template (Additional file 4: Table S3, Fig. 4). Interestingly, using FoHeli1-specific primers, a PCR product of 800 bp was amplified. The sequence of this PCR product corresponds to a FoHeli1 with joined ends (Fig. 4d) and does not occur in the assembled genome. Moreover, the intensity of the PCR product obtained using this primer pair is low compared to that of the PCR products obtained using the other, ‘genomic’ primer pairs (Fig. 4c), which is to be expected if its template is low-abundance, extra-chromosomal circular DNA. Notably, no PCR products corresponding to a FoHeli with joined ends were obtained using outward directed primer pairs specific for the subgroups that were more diverged in sequence, and therefore predicted to be non-active, FoHeli2—FoHeli5.

We tried to confirm the presence of circular Helitrons through multiply-primed Rolling Circle Amplification (RCA) [46] in which circular templates are overamplified with respect to the linear ‘background’ genome into concatemers. These concatemers can then be digested with an enzyme and run on a gel to produce bands corresponding to the size of the circle. In our experiments we could not detect overamplification of FoHeli1 (Additional file 1: Figure S8), rather we observed bands that most likely correspond to mitochondrial DNA. This can be explained by the extremely low abundance of FoHeli1 circles—caught in the act during DNA isolation- in the genomic DNA. They could easily have been outcompeted by the large amount of mitochondrial DNA during RCA and thus not have been amplified to such an extent that it would result in observable bands. However, when we isolated ~6–7 kb fragments from the gel (corresponding to the size of FoHeli1), we were able to obtain amplicons that correspond to FoHeli1 with closed ends (Additional file 1: Figure S8), thus confirming our previous result.

In *M. lucifugus, Drosophila*, Rice and Maize, multiple tandem insertions of Helitrons or Helitron-derived elements have been reported [3, 6, 7, 24]. We observed one case in which a FoHeli4 was inserted 2 bp upstream of the 5’ terminal partial sequence another FoHeli4 element (Fig. 3). We considered the possibility that the PCR product was amplified from a tandem insertion of FoHeli1 in the Fol4287 genome that was not assembled correctly, rather than a circular intermediate.

We mapped Illumina sequencing reads of Fol4287 from three different libraries with distinct insert sizes to a constructed sequence corresponding to a tandem insertion of FoHeli1 (see Additional file 1: Figures S9 and S10 for more detail). The mate-pair library, with the largest insert size (5 kb), contained one read that spanned the junction of the two FoHeli1 copies, and a few paired reads that were mapped on either side of this junction. However, mate-pair libraries tend to suffer from contamination with paired-end and overlapping reads and we found no reads either spanning the junction or crossing the junction as pairs in the other two libraries. Hence we conclude that it is unlikely that FoHeli1 occurs as a tandem insertion in Fol4287 (Additional file 1: Figure S9 and Figure S10).

### Some FoHelis have multiple 5’ termini

Some Helitrons, including non-autonomous Helitrons and partial Helitron copies, possess multiple termini (Additional file 6: Table S6). Interestingly, different genomes harbor different ‘versions’ of multiple termini. For example, *F. oxysporum* f.sp. *vasinfectum* contains partial copies in which the first 73 nucleotides of FoHeli1 are repeated once, whereas copies in *F. oxysporum* f.sp. *conglutinans* PHW808 repeat the first 85 nucleotides (Additional file 6: Table S6). *F. oxysporum* f.sp. *cubense* II5 contains partial copies of FoHeli1 that contain the first 31 or 65 bp of the 5’ terminus, and combinations thereof. Two tomato infecting strains, *F. oxysporum* f.sp. *lycopersici* MN25 and Fol4287, contain partial Helitron copies in which the first 31 nucleotides are duplicated. Helitrons with two or more 5’ termini are found in different locations in the genome. Multiple sequence alignments of these termini, including flanking genomic sequences, show a sharp decline in similarity at Helitron borders, indicating that these copies arose via transposition rather than via segmental duplication (Additional file 1: Figure S11).

### Helitrons are found in close proximity to pathogenicity-related genes

As mentioned above, Helitrons are potentially able to capture (parts of) genes and combine them into new transcripts transcripts [6, 18, 20, 25, 26]. Gene capture by Helitrons occurs very frequently in maize, but has rarely been observed to that extent in other species. Hence, pervasive gene capture is not a universal property of Helitrons. We investigated whether genes could have been captured by FoHelis. To this end, we compared all full-length putative autonomous and non-autonomous elements to NCBI’s non-redundant nucleotide database, removing hits that were likely to be misannotated Helitrons rather than captured host genes. This resulted in a list of 27 putative gene capture events, most of which are hypothetical proteins identified in the fungus *Metarhizium* (Additional file 7: Table S7).

Although we didn’t find evidence that gene capture by FoHelis plays an important role in FOSC evolution, we did note that some Helitrons are located in very close proximity to genes that have been implicated in pathogenicity in FOSC. For example, in the Arabidopsis-infecting strain *Fusarium oxysporum* Fo5176, a Helitron is found upstream of both *SIX9a* and *SIX9b*, homologs of the effector gene *SIX9* (Secreted In Xylem 9) encoding a protein identified in the xylem sap of tomato plants infected with Fol4287 [47]. A partial copy of a FoHeli2 is found 167 bp upstream from SIX9a, and a partial copy (the last 34 residues) of FoHeli1 is found 412 bp from SIX9b. Moreover, we find in the same strain a partial copy of a FoHeli1 or FoHeliNA2 located ~2 kb from a homolog of *SIX1* (Secreted In Xylem 1) of Fol4287 [47, 48]. Additionally, in the reference strain Fol4287, FoHeli1.6 is located 251 bp upstream from *SIX6* (Secreted In Xylem 6). In a race 1 tomato-infecting strain (Fol004) a FoHeli1 is located 156 bp upstream from a gene for a secreted oxidoreductase (ORX1-like) protein (AKC01502.1). Finally, in a melon-infecting isolate (Mel02010), we find a partial copy of a FoHeli1 located 476 bp upstream from a predicted argininosuccinate lyase gene (*ARG1*, AB045736.1). Deletion of *ARG1* leads to a reduction in virulence [49]. All partial copies in these examples lie on the border of the sequence that was submitted to GenBank, hence they could very well be complete copies that have either not been sequenced or not correctly assembled. Ectopic recombination between (almost) identical Helitron sequences can result in deletion of genomic regions. If these regions contain genes that are involved in infection, this may contribute to changes in virulence [50].

### FoHeli elements cluster phylogenetically with Helitron2 proteins

**Fig. 5:**
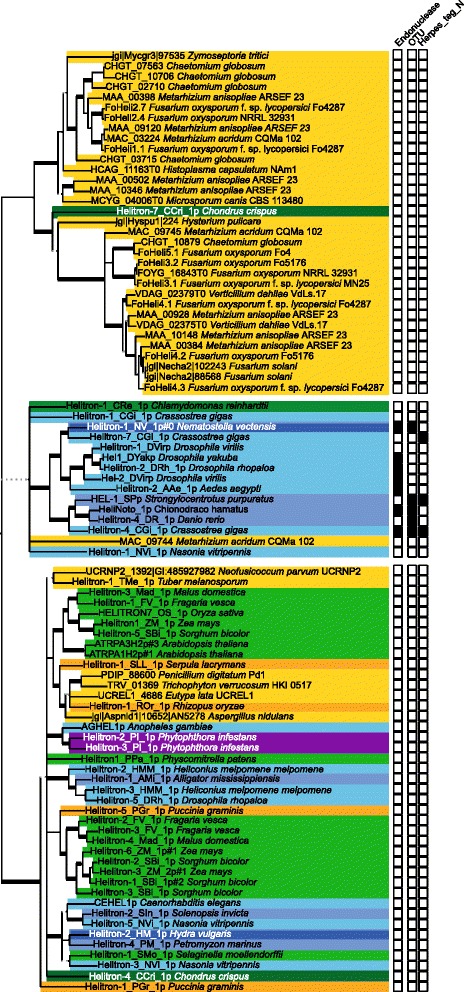
Phylogenetic relationships and domain architecture of FOSC and other fungal Helitrons, Helentrons and canonical Helitrons. Phylogeny based on a multiple sequence alignment of known Helitrons and Helentrons from RepBase, and a set of fungal Helitrons detected by scanning fungal proteomes for proteins with a Hel-Rep domain architecture. Due to space constraints, we do not show all proteins included in the phylogeny but selected a subset that represents the full tree as depicted in Additional file 1: Figure S13. We inferred a 100 bootstrap replicates: thick branches have a bootstrap value of 100 and grey branches a bootstrap value < 70. Branches with bootstrap values < 50 have been removed. Background color of leaves indicates taxonomy: yellow - Fungi, light-blue insects, blue – other Animals, green - Plants, dark green - Red Algae, purple – Oomycetes. All proteins included in this tree have a Hel and a Rep domain. The Helentrons that posses an endonuclease domain, often combined with a OTU domain, form a distinct group

The termini of FoHelis suggest that they belong to the Helitron2 variant [2]. To test this, we compiled a set of Helitron protein sequences extracted from RepBase [51] and Helitron2 sequences described in [2]. We also wanted to know how FoHelis relate to Helitrons found in relatively closely related fungi, hence we searched 102 Pezizomycotina proteomes for proteins with a Rep-Hel domain architecture. We predicted 45 proteins in 16 Pezizomycotina species to be putative Helitrons and added those to our dataset. We inferred a phylogeny and find that FoHelis and most fungal Helitrons group with Helentrons and other Helitron2 elements with high bootstrap support (Fig. 5, Additional file 1: Figure S12, Figure S13). This suggests that many fungal Helitrons have non-canonical termini. We find no fungal putative Helitrons that contain an endonuclease domain, the hallmark domain of Helentrons.

### Conservation of terminal features: FoHeli-like termini in other fungi

FoHelis share several features with members of the Helitron2 variant, but none of these members have the exact same terminal sequences as FoHelis [2]. To determine to what extent the exact termini are FOSC-specific we searched a database of 102 Pezizomycotina genomes for Helitrons with FoHeli-like termini (Additional file 8: Table S8). For each subgroup, we find at least one sequence outside FOSC that possesses FoHeli termini (Additional file 9: Table S9). The species in which we find completely conserved FoHelis (i.e. including termini) corresponds to what we would expect given the tree presented above: FoHeli1 is present in *Metarhizium anisopliae* ARSEF 23 (currently corrected to *Metarhizium robertsii)*, FoHeli4 in *Verticillium dahliae VdLs.17* and FoHeli5 in *Chaetomium globosum. Fusarium solani* has all FoHeli subgroups except FoHeli5. In *Metarhizium acridum*, we only find 3’ termini, except for one case in which we observe three Helitron copies in tandem. Either a Helitron was inserted into the 5’ end of another Helitron twice (MAC_03224 and MAC_3225 in Additional file 1: Figures S12 and S13), or this is the result of rolling circle replication of single stranded circular DNA. Finally, we find FoHeli2 in *F. acuminatum*, and FoHeli2, FoHeli3 and FoHeli4 in *F. virguliforme*, genome sequences for which annotations are not publicly available.

Interestingly, FoHeli1 sequences in *F. solani* bear hallmarks of Repeat Induced Point (RIP) mutation with a more than 3-fold increase in CpA to TpA and TpG to TpA mutations compared to other G- > A and C- > T mutations (Additional file 1: Figure S14). RIP is hypothesized to function as a genome defence mechanism against duplicated genes and TEs and RIP can at least partially explain why we do not find a large number of proteins with a Hel-Rep domain architecture in *F. solani* [52, 53].

**Fig. 6:**
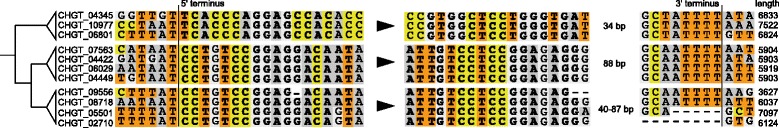
Termini of Helitrons in *Chaetomium globosum* exemplify conservation of 3’ terminal sequences. We determined the termini for two groups of *Chaetomium globosum* Helitrons that group together with FoHeli1 and FoHeli2 in the tree in Fig. 5, Additional file 1: Figure S12 and Figure S13. Terminal inverted repeats (TIRs) are in bold. In contrast to the ATIRs in FoHeli1 and FoHeli2, the ATIRs of these Helitrons are not hairpins. The sequence of the 3’ termini closely resembles those of FoHeli1 and FoHeli2, as they also end in ‘ATTTT’. Moreover, the bottom two subgroups possess imperfect palindromes overlapping their ATIRS

In the trees of Helitron sequences presented in Fig. 5, Additional file 1: Figure S13 and Figure S14, we find two clades of very similar Helitron sequences in *Chaetomium globosum* that neighbour the FoHeli1 and FoHeli2 clades. Yet we did not find FoHeli1- or FoHeli2-like termini in *Chaetomium globosum* using our blastn. To determine the termini for these Helitrons, we took the same approach as we did originally for FOSC Helitron sequences: we aligned the gene sequences including a large up- and downstream region and inspected these alignments to find termini for the Helitrons. We find that *C. globosum* Helitrons possess the 3’ terminus of FOSC Helitrons, including the ‘ATTTT’ and the inverted repeat, but do not have a hairpin at the 5’ end (Fig. 6). The 3’ terminus is more conserved than the 5’ terminus. Finally, *Chaetomium globosum* Helitrons, like FoHeli1 and FoHeli2, appear to insert between ‘TNAT’ and ‘A’, where ‘N’ denotes any nucleotide.

## Discussion

### Detection of non-canonical Helitrons

FoHelis likely represent a large reservoir of Pezizomycotina Helitrons that group phylogenetically with Helitron2 transposons, suggesting that most fungal Helitrons have non-canonical termini (Fig. 5). Indeed, we were able to confirm that Helitrons with FoHeli-like termini also occur in other fungi (Additional file 9: Table S9, Fig. 6). In the case of the FOSC, we would not have detected any Helitrons using conventional approaches based on termini or DNA sequences of canonical Helitrons [5, 18, 20, 39]. Our analyses of predicted putative Helitrons in other fungi suggests that the same may hold true for many other species [38, 40].

Another factor that hampered detection of FoHelis is their size. Genome assemblies based on second generation sequencing data are unlikely to include recently transposed elements of more than 5 kb [44, 45, 54]. Hence the repeat content of genomes that are assembled to different levels of completeness cannot be directly compared [40, 54]. Similarly, non-autonomous elements are often more abundant than their autonomous counterparts [1, 3], which can be explained by the intuitive assumption that shorter sequences are more efficiently transposed. On the other hand, non-autonomous elements are more likely to be assembled in one piece and therefore more easily detected. Hence we may have been overestimating their success as a parasite’s parasite. Improvements in genome assembly through the use of third and fourth generation sequencing technologies will allow us to better estimate and compare the TE repertoires of different genomes, to reconstruct the influence of transposons on genome evolution, but also to gain understanding on (co-) evolution of selfish elements in and across host genomes [19, 55–57].

### Self-insertion may have led to composite FoHelis

**Fig. 7:**
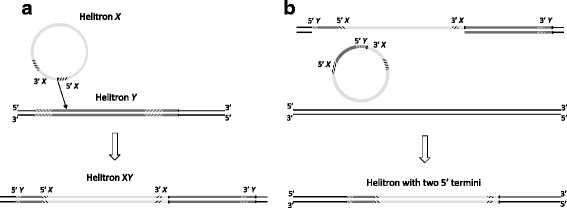
Multiple termini may arise by self-insertion. **a** Helitron *X* (*circle*) inserts into an other (non-autonomous) Helitron *Y*. Striped sections indicate the inverted repeats. **b** Excision leading to a chaemeric Helitron: Nicking by the Helitron transposase starts at the 5’ terminus of Helitron *Y*, the DNA is unwound until the transposase encounters a 3’ terminus, which is in this case the terminus of Helitron *X*. This chaemeric Helitron, containing two 5’ termini from two Helitron copies, can then be inserted somewhere else in the genome

**Fig. 8:**
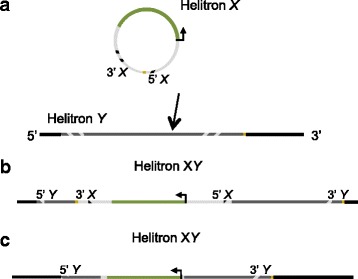
Reversal of ORF orientation after self-insertion. **a** Helitron *X* (*circle*) inserts into an other (non-autonomous) Helitron *Y* in opposite orientation. The green bar indicates the ORF, striped sections indicate the inverted repeats, yellow line the 3’ terminus. **b** The composite Helitron*XY*. **c** Deletion or mutation of internal termini yields a Helitron with a ORF in opposite orientation when compared to other Helitrons with similar termini

Self-insertion can lead to nested, composite or chimeric Helitrons [14, 24, 30]. In this study, we’ve found one example of a non-autonomous FoHeli nested into a putative autonomous one. Moreover, we’ve found a number of FoHelis in which multiple 5’ termini were combined with a single 3’ terminus. Typically, the 5’ sequence that is duplicated is short (<200 bp). These 5’ duplications may stem from nested FoHelis that result from self-insertion. If, during transposition, the transposase nicks the leftmost 5’ terminus of the nested Helitron, and continues to unwind the DNA until it encounters the first 3’ terminus, where it stops, it may transpose a FoHeli with two 5’ termini (Fig. 7). Reversal of ORF orientation may also stem from a composite or nested Helitron, in which one copy is inserted into the other in opposite orientation, after which the innermost set of termini is deleted or mutated and only the extreme termini are preserved (Fig. 8).

### Detection of circular intermediates

Results from this study indicate that FoHelis, like canonical Helitrons [14] transpose via a circular intermediate. However, we failed to amplify circular Helitrons using Rolling Circle Amplification, suggesting that we need additional preprocessing steps to enrich our genomic DNA samples for circular DNA other than from mitochondria to find circular Helitrons via this approach (e.g. as in [58, 59]). DNA isolation provides a snapshot of DNA content of a large number of cells and for a Helitron circle to be present, it has to transpose at that exact time. Therefore we expect very few circles to be present in one DNA sample and need extremely sensitive methods to detect them.

### Which FoHelis are still active in the FOSC?

In Fol4287, we’ve found two identical copies of FoHeli1 that, judging from their flanking sequences, arose through transposition rather than segmental duplication. Moreover, FoHeli1 is the subgroup we have found most at contig borders in Fol4287 and for which we found a PCR amplicon that could stem from a circular intermediate. This suggests that FoHeli1 is still active in the genome of Fol4287. The other subgroup that appeared to have had a strong impact on genome assembly is FoHeli2 that is predicted to occur in high copynumber in brassicaceae-infecting isolates. In contrast to the genome of Fol4287, the genomes of these isolates have not been assembled with the aid of an optical map. Improved assemblies, combined with detection of putative circular intermediates, may shed light on when FoHeli2 was active in these isolates.

## Conclusions

Helitrons have been studied for more than a decade, where the main focus has been on canonical Helitrons, or Helitron1, in plants and animals. Here we present the first study of non-canonical Helitron transposons in Pezizomycotina, shedding light on a Helitron variant in a subphylum that both have been relatively underrepresented in scientific literature on Helitrons. In FOSC, we’ve identified 2 groups with distinct terminal sequences. We presented data suggesting that FOSC Helitrons transpose via a circular intermediate, which has been shown for canonical Helitrons very recently [14]. Importantly, we found that most Pezizomycotina Helitrons are probably non-canonical. The information we provide here will aid in future identifications of Helitrons and thus contribute to a more accurate characterization of transposon repertoires, especially in Pezizomycotina.

## Methods

### Identification of putative autonomous Helitrons in FOSC

We select 35 genes from 10 different strains encoding proteins with a Rep (PF14214) and a Hel (PF05790) domain based on Pfam annotation for the 12 FOSC genomes provided by the Broad Institute [10, 13, 47, 60]. To detect additional copies that were excluded from the gene annotation, we used these 35 proteins as a query in a tblastn search to find homologous regions in the 12 FOSC genomes [61]: sequences were included if the alignment returned by BLAST covered at least 80% of the query with > = 80% identity. These sequences were extended up to 10 kb in each direction and annotated by FgenesH [62], an online program for gene prediction, using parameters of *Fusarium graminearum*. We determined the domain architecture for the proteins encoded in these predicted ORFs using hmmscan and the PfamA database, applying default inclusion thresholds. The genes that encode proteins with a Rep (PF14214) and a Hel (PF05790) domain, were considered putative autonomous Helitrons. In this way, we found 28 more Helitrons, bringing our total to 63 (Additional file 2: Table S1). These were subsequently used as queries to search for additional copies using blastn. We found no additional full-length copies. In total, we retrieved 63 Helitron protein sequences in the FOSC (Fig. 1, Additional file 2: Table S1).

### Phylogenetic analyses of FOSC Helitrons

To assess how these 63 FOSC Helitrons are clustered into subgroups, we aligned protein sequences using prank [63] with default settings, trimmed the multiple sequence alignment with trimAl –strictplus [64] and inferred a tree using PhyML v3.0 [65] with 4 substitution rate categories, estimated proportion of invariable sites and gamma distribution. We run PhyML once to produce bootstrap support (100 bootstraps) and once with aLRT branch support (SH-like). For the tree depicted in Fig. 1, branches that have aLRT-support < 0.9 and/or bootstrap support < 80 were collapsed using a custom python script implementing ete2 [66]. We found that FOSC Helitrons can be divided into 5 subgroups, here designated FoHeli1-FoHeli5 (Fig. 1).

### Identification of Helitron termini

If different copies of a transposable element arose through transposition (as opposed to segmental duplication), sequence similarity between the copies extends up to the termini of the transposable elements, but not further. We use this to identify termini for FOSC Helitrons. For each FoHeli subgroup, we add 1–7 kb of flanking sequences to the predicted gene sequences, if possible, i.e. if the Helitron is not to close to the border of a (super)contig. We align these sequences using Clustal Omega [47] and manually inspect alignments to find the regions where the sequences change from dissimilar to very similar (5’ terminus) or from very similar to dissimilar (3’ terminus). We use this approach to identify termini in *Chaetomium globosum* as well (Fig. 6). To identify FOSC termini in other fungal species we queried a database of 102 Pezizomycotina genomes with the DNA sequences of FoHeli full elements. We combined all partial hits of the same FoHeli query that are located within close distance (<3 kb), aligned the corresponding region with the query and inspected the alignment to determine whether FoHeli termini were indeed present.

For each subgroup, we reconstructed pre-insertion sites by concatenating 500 bp 5’ flanking sequence of the FoHeli with 500 bp of 3’ flanking sequence of the FoHeli. In some cases the FoHeli resides closer than 500 bp to a supercontig border, then we took as much flanking sequence as we could. We use blastn to search for these pre-insertion sites within the 12 FOSC genomes. We used a custom python script to extract the sequences of BLAST hits that bridge the two flanking sequences, write these sequences to a fastafile and align these with Clustal Omega to confirm the termini we inferred are correct.

### Estimation of FoHeli copynumber from partial hits

We expected that the number of Helitrons we found in our initial survey [63] is an underestimate of the real copynumber as a result of e.g. gaps in the genome assembly or regions of high divergence within Helitrons. We search the 12 FOSC genomes using megablast, with the 41 FOSC terminus-to-terminus Helitron sequences as queries, each with 100 bp of flanking sequence. The resulting blast output was parsed using a Python script. We only considered hits that start after the first 90 and end before the last 90 bp.

Due to low complexity or gaps between the contigs that are represented by ‘N’s, BLAST may produce multiple alignments of a query (sub)sequence and a subject (sub)sequence. To avoid overestimating the number of partial hits because of this, we first merged hits that were less than 200 bp apart in the query, but for which the overlap in the query was <50 bp (to ensure that individual hits represent different parts of the query), and less than 2000 bp apart in the subject (scaffold) sequence, assuming that these multiple hits represent one putative Helitron sequence on the supercontig. Moreover, we merged hits that represented multiple termini.

### Identification of putative gene capture events

Helitrons are well-known for their ability to capture (parts of) genes [4, 6, 7, 14–22]. To determine the extent of gene capture in FOSC Helitrons, we search NCBIs non-redundant nucleotide database (nr/nt) using 48 full-length FOSC Helitrons. We use a custom python script to query the Entrez database with the Genbank Identifiers returned by the BLAST search. We select hits that contain a coding sequence and find the corresponding protein sequence. We infer domain architectures for these protein sequences using hmmscan from the hmmer3 package [67] and the PfamA database (Pfam 27.0) [68] and select proteins that do not contain a Helitron-like_N (Rep) or PIF1 (Hel) domain. We thus obtain a list of 27 genes that have been (partially) captured by a FOSC Helitron.

### DNA isolation, PCR analysis and sequencing

We use PCR to detect circular intermediates of FOSC Helitrons (Fig. 4). Fungal genomic DNA (gDNA) was extracted using the following method: a patch of mycelium was scraped from the margin of a colony and suspended in 400 μl Tris-EDTA buffer (1 M Tris pH 8, 0.5 M EDTA pH 8) together with 300 μl phenol:chloroform (1:1) and approximately 300 μl glass beads (212–300 μm). Cells were mechanically disrupted in a tissuelyser for 30 s. The supernatant (150 μl) was collected after centrifugation (5 min) at maximum speed and mixed with equal volume of chloroform. Again, the supernatant (100 μl) was collected after vortexing and centrifugation (5 min) and kept in -20 °C for further use. 1 μl of genomic DNA was used for PCR experiments. Primers used for amplification of the FoHeli joined ends are listed in Additional file 1: Table S4. The amplified products were resolved electrophoretically in a 1% agarose gel. PCR products were sequenced and analyzed using Seqbuilder.

### Rolling circle amplification and downstream analyses

Rolling circle amplification was performed on 80 ng Fol4287, 250 ng Fol4287, 80 ng Fol029, 80 ng Fo5176, 80 ng Fo47, 80 ng of Fom001 genomic DNA and a 5169 bp plasmid spiked into 80 ng of Fo47 genomic DNA, as described by [46] using phi29 DNA polymerase (#EP0091, Thermo Scientific), inorganic pyrophosphatase (#EF0221, Thermo Scientific) and exo-resistant random primers (#S0181, Thermo Scientific) in a 12.5 h, 20 μL reaction at 30 °C (Additional file 1: Figure S7). The reaction was stopped by elevating the temperature to 65 °C for 10 min. Subsequently, 5 μL of the amplification product was digested with Acc65I, XhoI or EcoRV for 3 h and run on a 1% agarose gel. A band of the expected size (~6–7 kb) was observed and extracted from the gel using a QIAquick Gel Extraction Kit according to the manufacturer’s protocol. 1 μL of the 6–7 kb fragment was used for a regular PCR using primer pairs distributed over the length of FoHeli1 (Additional file 1: Figure S8).

### Phylogenetic analyses of FOSC, pezizomycotina and known Helitrons

For the phylogenetic analyses including known Helitron1 and Helitron2 from RepBase (version 19.11), we used custom Python scripts to parse RepBase files for protein sequences of Helitrons. In addition, we obtained Helitron2 sequences described in [2] from the authors. These include all proteins that reside within Helitron termini, hence also e.g. replication protein A (RPA)-like proteins. We predicted domain architecture for these proteins using hmmscan from the hmmer3 package [67] and the PfamA database (Pfam 27.0) [68]. We used custom Python scripts and manual curation to determine the final domain architecture of individual proteins: in case of overlapping domain predictions (mostly PIF1 domains that also matched AAA domains), we kept the domain with the highest score (PIF1), or, in cases in which predictions likely correspond to the same domain, we merged overlapping regions that also overlapped in a similar fashion (e.g. no inversions) in the hmm model. In further analyses, we only include protein sequences that contain a Rep and a Hel domain (PF14214 and PF05970). We use hmmsearch from the hmmer3 package [67] with PF14214 and PF05970 to scan all Pezizomycotina proteomes in our dataset (Additional file 8: Table S8) for proteins that contain both these domains. We constructed two different multiple sequence alignments for this set of proteins. First, we cut out Rep and Hel domains from each protein, removed identical sequences, aligned the domain sequences using hmmalign and concatenated the alignments of both domains. Second, we aligned full protein sequences using Clustal Omega with default settings [69]. We then trimmed this alignment with trimAl (−gappyout), removed identical sequences and used RaxML to infer the phylogeny (options: −f a -N 100 -m PROTGAMMAIWAG -x 1234567 -p 123 (Additional file 1: Figure S12 and Figure S13 and Fig. 5) [70]. Figure 5 shows the Clustal Omega tree, where branches with a bootstrap support of less than 50 trees were collapsed.

For all the putative Helitron proteins in the these trees we predicted whether any domain other than the Rep and Hel domain was present using hmmscan from the hmmer3 package and the PfamA database (Pfam 27.0) [67, 68]. The domain architecture of proteins is summarized in Fig. 5, and shown more elaborately in Additional file 1: Figure S12 and Figure S13. Many putative Helitrons have an N-terminal Helicase domain that is classified in Pfam as either a Herpes_Helicase, an UvrD_C_2 or a Viral_helicase_1 domain (Additional file 1: Figure S12 and Figure S13). However, when we overlay the conserved Hel motifs I-VI in FoHelis (Additional file 1: Figure S2) to the automated Pfam domain prediction, we find that only motifs I until IV/V lie in the predicted Hel domain, whereas the other two motifs (V and VI) lie in the predicted N-terminal Helicase. This means that the automated prediction of both the Hel and the N-terminal Helicase is probably incorrect and that the predicted N-terminal Helicase domains are actually part of the Hel domain.

## Additional files

Additional file 1: Supplemental file with Figure S1,S2 and S3 that show conserved motifs in FoHeli protein sequences, Figure S4 that shows a dotplot of chromosome 3 and chromosome 6, with FoHelis on the diagonal, Table S4 that contains the primers in this study, Figure S5 that shows an alignment of non-autonomous FoHeliNA1 with FoHeli1, Figure S6 that shows an alignment of non-autonomous FoHeliNA2 with FoHeli1, Figure S7 that shows a gel with the product of RCAs, digested with different enzymes, Figure S8 that shows results of PCR amplification of a 6-7 kb band from Figure S7, Figure S9 and S10 shows the results of mapping unpaired and paired reads on a constructed tandem FoHeli1, Figure S11 shows alignments of FoHelis with multiple termini and their flanking regions, Figure S12 and S13 show phylogenetic trees of FoHelis with other predicted Helitrons and Figure S14 ﻿shows putative﻿ RIP mutations in FoHeli1 homologs in *F. solani*. (PDF 17380 kb)
Additional file 2: Table S1: FoHelis found in this study: position on the genome, start and end sequences, position of the predicted ORFs. (XLSX 46 kb)
Additional file 3: Table S2, additional ORFs predicted within FoHeli termini. (XLSX 38 kb)
Additional file 4: Table S3: non-autonomous FoHelis. (XLSX 47 kb)
Additional file 5: Table S5: Copynumber estimate based on partial hits. (XLSX 272 kb)
Additional file 6: Table S6: FoHelis with multiple 5' termini. (XLSX 65 kb)
Additional file 7: Table S7: Putative gene capture events. (XLSX 123 kb)
Additional file 8: Table S8: Number of protiens with a Rep and/or Hel domain found in queried species. (XLSX 22 kb)
Additional file 9: Table S9: FoHeli-like sequences found in other species. (XLSX 39 kb)
