# Supplementary material for: Non-canonical Helitrons in Fusarium oxysporum
Source: Mob DNA. 2016 Dec 9;7:27. doi: 10.1186/s13100-016-0083-7 (PMC5148889; doi:10.1186/s13100-016-0083-7)
Supplement: Additional file 1: — Supplemental file with Figure S1,S2 and S3 that show conserved motifs in FoHeli protein sequences, Figure S4 that shows a dotplot of chromosome 3 and chromosome 6, with FoHelis on the diagonal, Table S4 that contains the primers in this study, Figure S5 that shows an alignment of non-autonomous FoHeliNA1 with FoHeli1, Figure S6 that shows an alignment of non-autonomous FoHeliNA2 with FoHeli1, Figure S7 that shows a gel with the product of RCAs, digested with different enzymes, Figure S8 that shows results of PCR amplification of a 6-7 kb band from Figure S7, Figure S9 and S10 shows the results of mapping unpaired and paired reads on a constructed tandem FoHeli1, Figure S11 shows alignments of FoHelis with multiple termini and their flanking regions, Figure S12 and S13 show phylogenetic trees of FoHelis with other predicted Helitrons and Figure S14 ﻿shows putative﻿ RIP mutations in FoHeli1 homologs in F. solani. (PDF 17380 kb) [file 13100_2016_83_MOESM1_ESM.pdf]

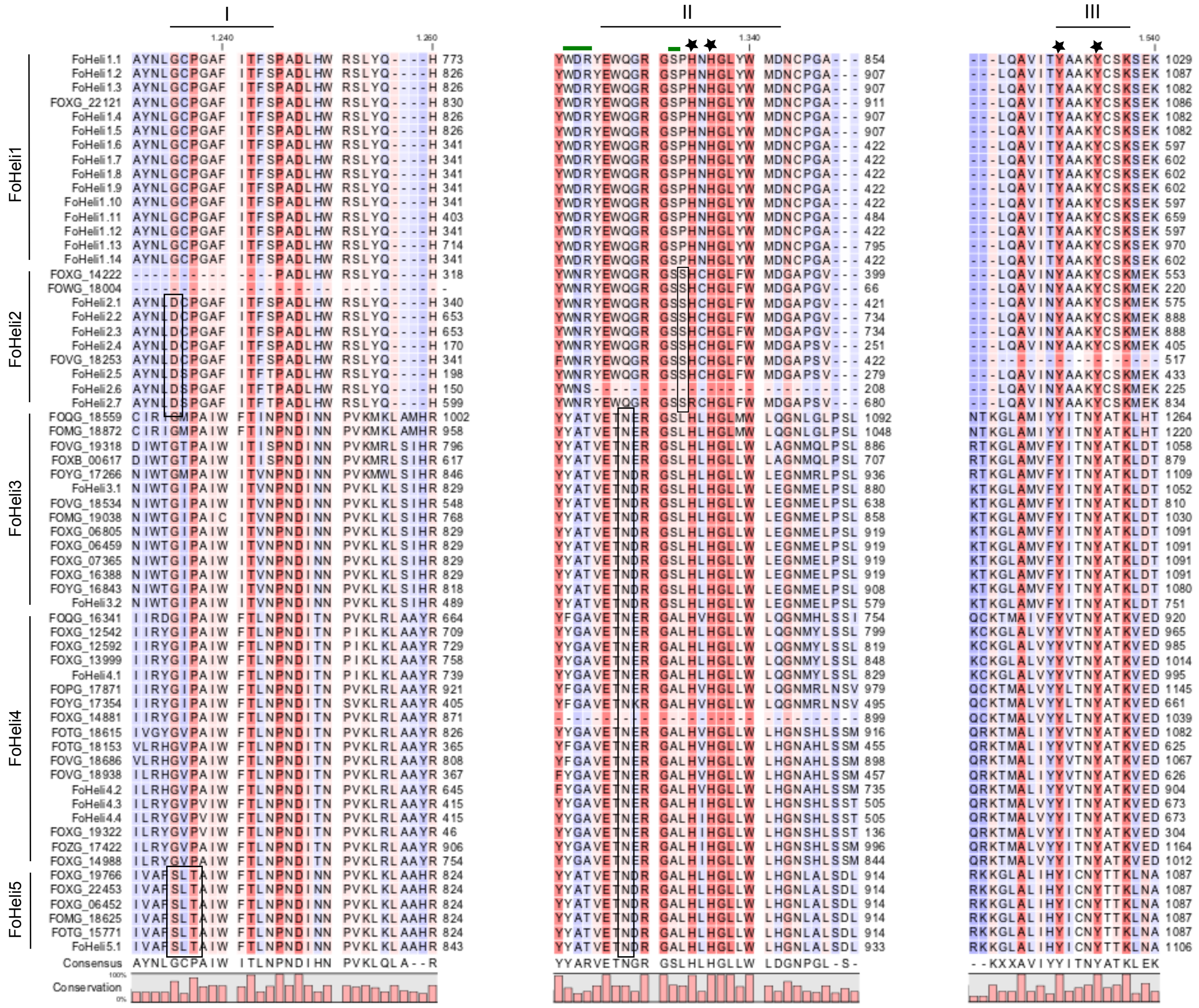

Figure S1: Important functional motifs in the Rep domain are conserved in most FoHelis. Here we show cutouts from a multiple sequence alignment of FoHeli proteins, where the background colouring indicates conservation, going from blue (not conserved) to red (conserved). For each cutout, the index of the rightmost residue in the unaligned protein sequence is indicated on the right of the sequence. We put black boxes around residues that are part of conserved motifs, yet differ per FoHeli subgroup.

Three motifs are evolutionary conserved in the Rep domain (Koonin and Corbalenya 1993; Koonin and Ilyina 1993; Fairman-Williams et al. 2010), indicating that they are important residues for the structure and function of this domain. The first motifs is defined as ‘GxPxh[F,Y]h[T/S]hs’ where x means ‘any amino acid’ and h means ‘any hydrophobic amonio acid’.

The second motif is defined as ‘ExQxRxxx[P,L]HxHhhh[W,F]h’, and again FoHeli2 has substituted one of the normally conserved residues for an amino acid with similar properties, in this case P for S, again both small. The motifs that distinguish Helentrons from Helitrons, ‘axp’ (aromatic, any residue, polar) and ‘S’ are indicated by green bars on top of motif II. FoHeli1, FoHeli2 and FoHeli3 share these motifs.

The histidine and tyrosine catalytic residues in motif II and III are indicated with stars on top of the alignment.

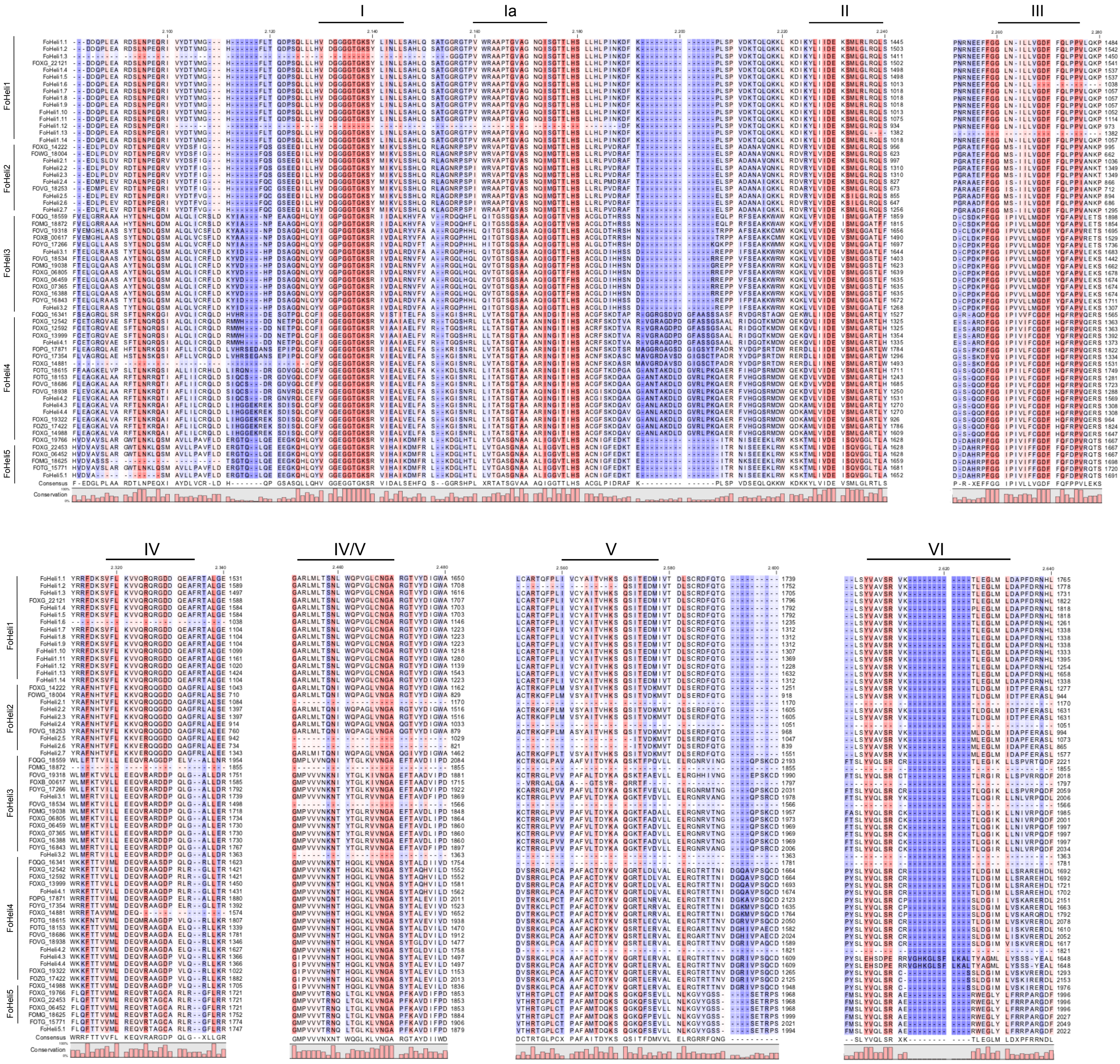

Figure S2: Important functional motifs in the Hel domain are conserved in most FoHelis.
Here we show cutouts from a multiple sequence alignment of FoHeli proteins, where the background colouring indicates conservation, going from blue (not conserved) to red (conserved). For each cutout, the index of the rightmost residue in the unaligned protein sequence is indicated on the right of the sequence.

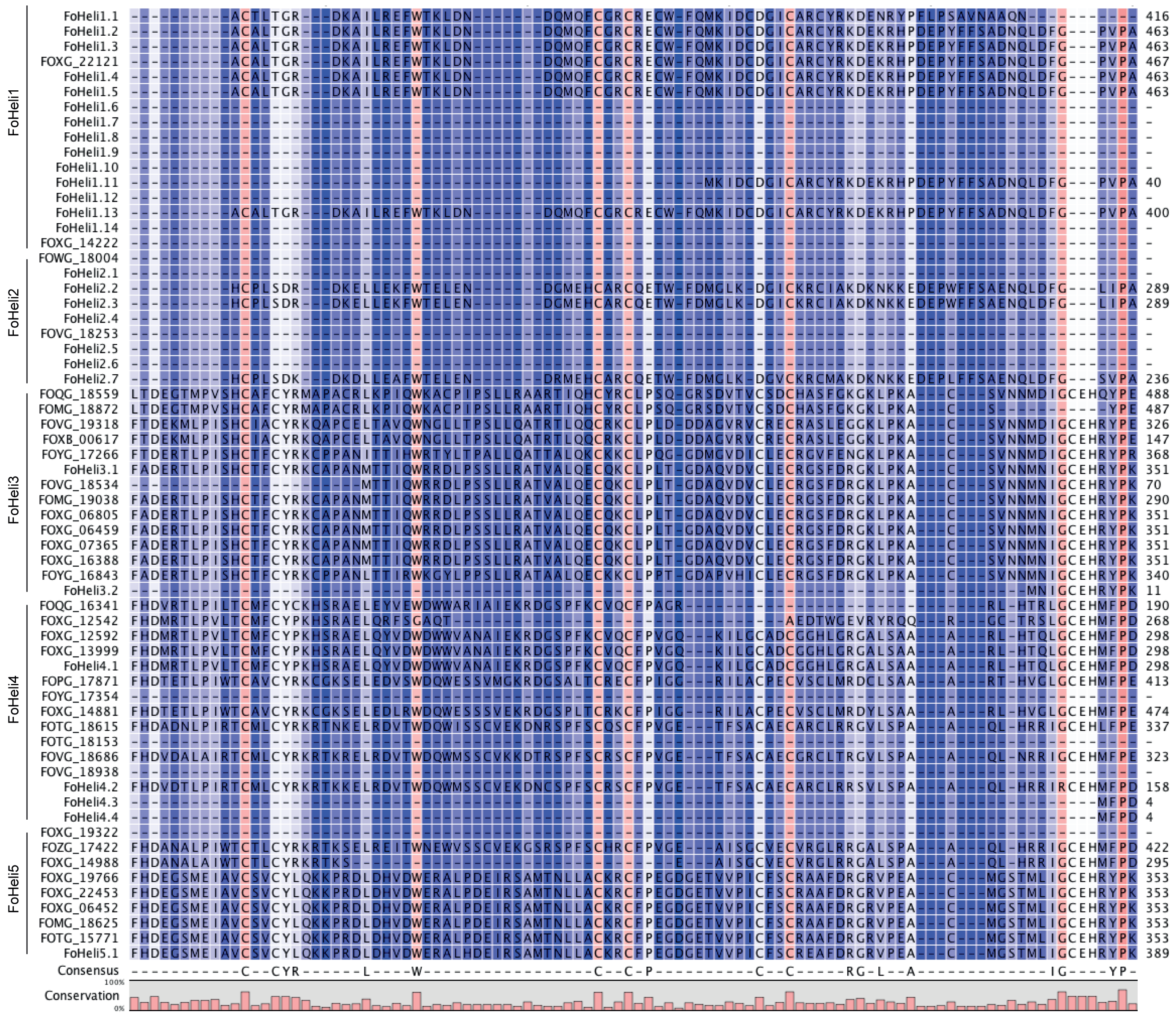

Figure S3: N-terminal zinc finger-like motif
Here we show a fragment from a multiple sequence alignment of FoHeli proteins, where the background colouring indicates conservation, going from blue (not conserved) to red (conserved). The index of the rightmost residue in the unaligned protein sequence is indicated on the right of the sequence. We have not identifed any known Pfam domain in this regions, but the spacing of cysteine residues is similar to that found in zinc finger DNA binding motifs, suggesting that the N-terminus is involved in DNA binding. This motif is not present in all predicted protein sequences, which may partly be contributed to erroneous gene prediction.

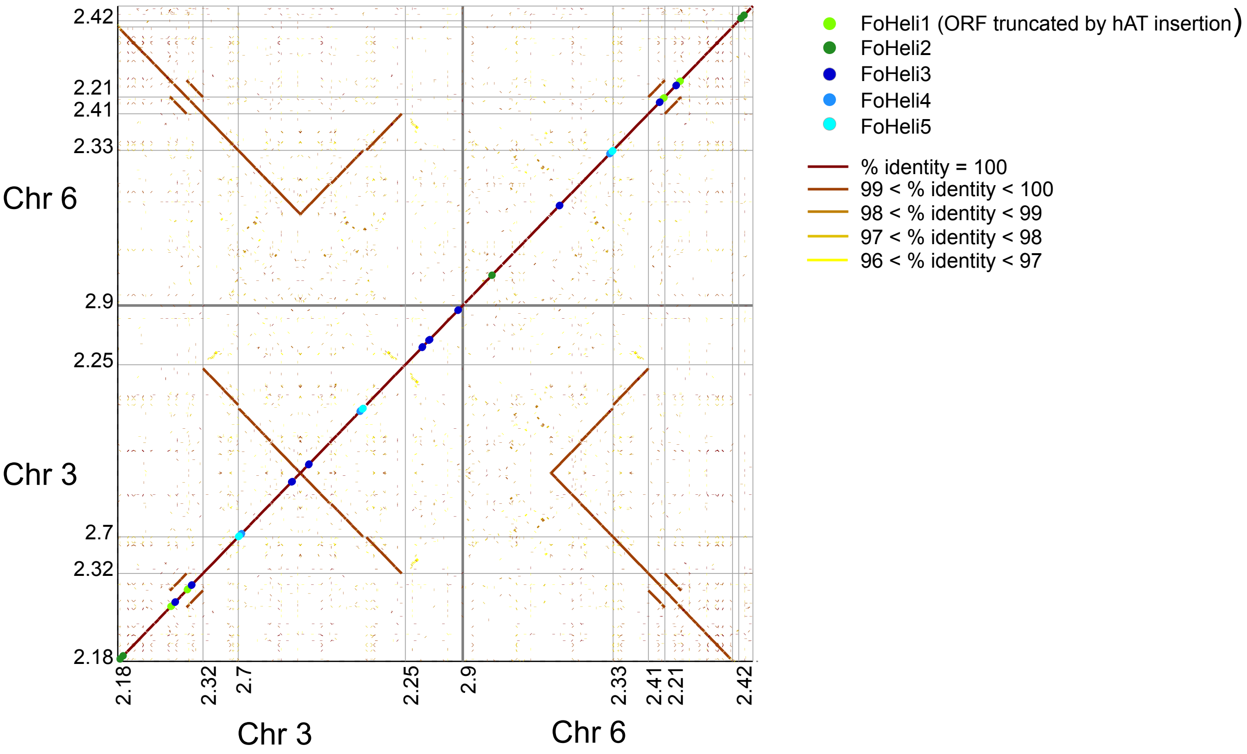

Figure S4: Some nearly identical copies of FoHeli are due to recent large-scale segmental duplications, rather than recent transposition events.

Figure 1 in the main text shows that the reference genome Fol4287 contains several nearly identical copies of FoHeli2-FoHeli5 sequences. These copies arose via recent duplications of large segments on chromosome 3 and 6.

We aligned the supercontigs that were aligned to chromosome 3 and 6 based on optical mapping (Ma et al. 2010) using MUMmer (nucmer –maxmatch) (Delcher et al. 2002) and plot aligned segments longer than 500bp, coloured according to the %identity of the alignment. The location of FoHelis on the chromosomes is indicated on the diagonal, coloured according to the subgroup the Helitorn belongs to. The FoHeli1 copies are the ones that have a hAT insertion and were identified when searching for non-autonomous elements and were thus not included in Figure 1 in the main text.

No FoHeli is located exactly at the border of any of the duplications and rearrangments, hence there is no indication that these occurred due to homologous recombination between FoHeli copies.

Primer number	Sequence	Target sequence*
4297	TGCCTCTTTGCTCTGAAGG	FoHeli1.12-5' end (2)
4242	ACAAGTCACAAAGCATCAC	FoHeli1.12-3' end (3)
4303	ACTTCCCAGTGACAAACGC	616 bp upstream of FoHeli1.12 (1)
4340	ACAACGGGGACATTGATGGC	1382 bp downstream of FoHeli1.12 (4)
5520	TACAGTCGAGACAGTTAACC	FoHeli2 FOXG_14222-5' end (2)
5521	TTCACAAGTCACAGAGCATC	FoHeli2 FOXG_14222-3' end (3)
5538	TGGATTCCTGTTCTTTCATC	446 bp upstream of FoHeli2 FOXG_14222 (1)
5537	TGATGTGCAGCTACGGACTC	622 bp downstream of FoHeli2 FOXG_14222 (4)
5522	TACGGTATTCGTATCTGTCG	FoHeli3 FOXG_06459-3' end (2)
5524	ATGTGATGACCTGATTATCC	FoHeli3 FOXG_06459-5' end (3)
5539	AAGGTACACTTCGTTGACG	397 bp upstream of FoHeli3 FOXG_06459 (1)
5540	AGTTGGCCGGTTGAGCAGAG	310 bp downstream of FoHeli3 FOXG_06459 (4)
5525	AGCCTCTCTTCTCGGATGAC	FoHeli4 FOXG_12592-5' end (2)
5526	TTCAGTTCGAAGGAAGAAGG	FoHeli4 FOXG_12592-3' end (3)
5542	AGATGTCAACGTCTTCAATC	550 bp upstream of FoHeli4 FOXG_12592 (1)
5541	TCATCAACATCACGGCAATG	824 bp downstream of FoHeli4 FOXG_12592 (4)
5527	TCAGCTACCTCTTCTTTCC	FoHeli4.3 5' end (2)
5528	TGGAGCAACTGAGCAATAGG	FoHeli4.3 3' end (3)
5544	TACTGCAATGCGGTCAATTC	385 bp upstream of FoHeli4.3 (1)
5543	ACAAGTTCGAGGAATCTGAG	577 bp downstream of FoHeli4.3 (4)
5529	TGGACATTACGTGTATTCGG	FoHeli5 FOXG_06452-5' end (2)
5530	AATGTCGCATGGCTGTTCAG	FoHeli5 FOXG_06452-3' end (3)
5546	TCATGTCCCAATTCAGAAGTG	698 bp upstream of FoHeli5 FOXG_06452 (1)
5545	TCTATGCAACTCTCGCTTGAC	588 bp downstream of FoHeli5 FOXG_06452 (4)

*Numbers in brackets correspond to primers as shown in Figure 3A

Table S4. Primers used in this study
These primers were used to detect FOSC Helitrons with closed ends. We used two sets of primers for FoHeli4 because it more diverse than the other groups.

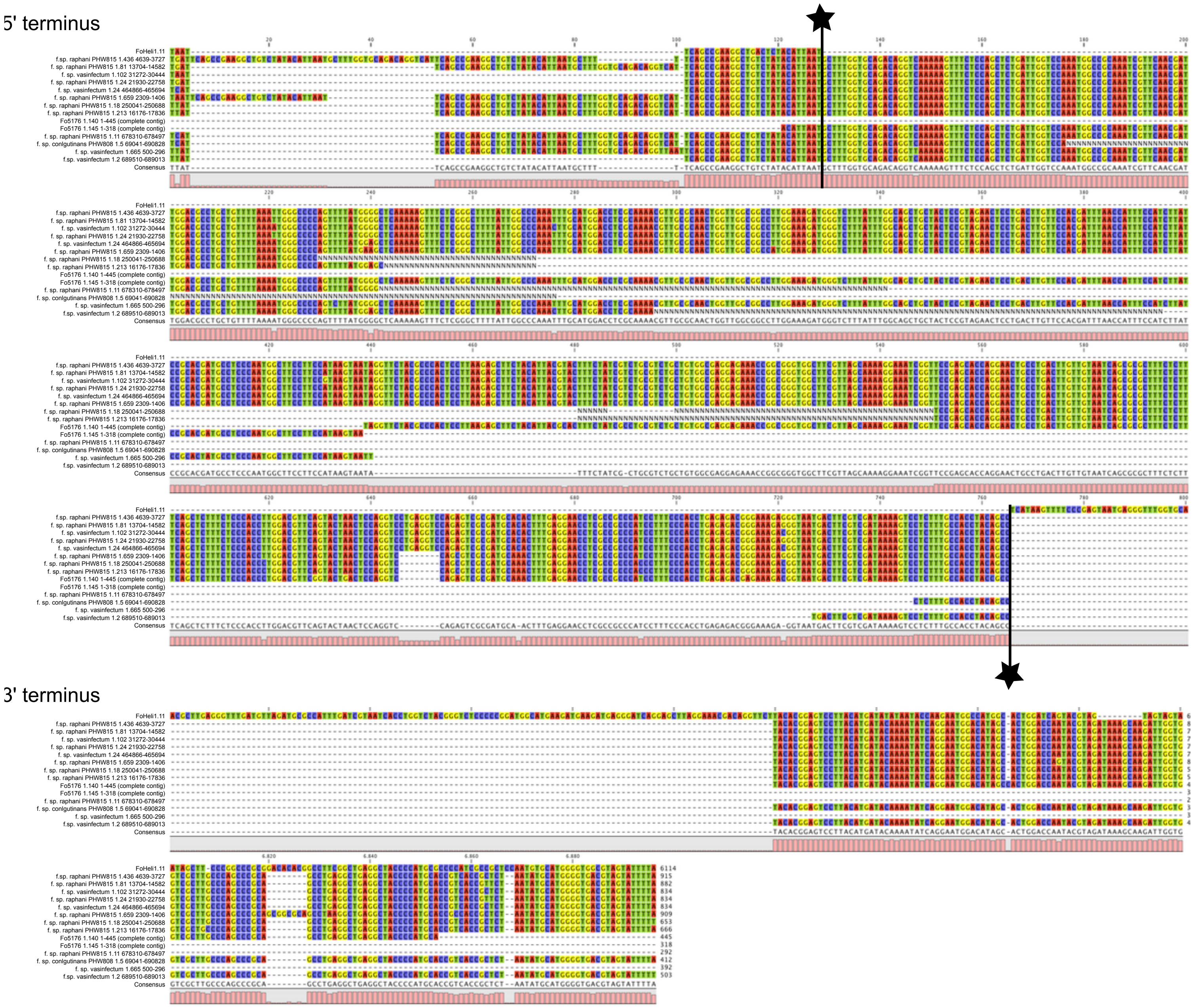

Figure S5: Alignment of non-autonomous element FoHeliNA1 with FoHeli1 shows it derived from FoHeli1.

Multiple sequence alignments of the 5’ (A) and 3’ termini (B) of 6 FoHeliNA1 sequences (see Table S2 for exact coordinates of these sequences) and FoHeli1.1. Nucleotides are coloured as follows: A red, T green, G yellow, C blue. The first ~27bp of FoHeliNA1 are ~90% identical with FoHeli1. FoHeliNA1 contains 628 bp of extra sequence (between black stars) that is not homologous to any Helitron sequence in FOSC. We tried to identify homologous sequences using BLAST searches against NCBI nr/nt database, but did not retrieve significantly similar sequences.

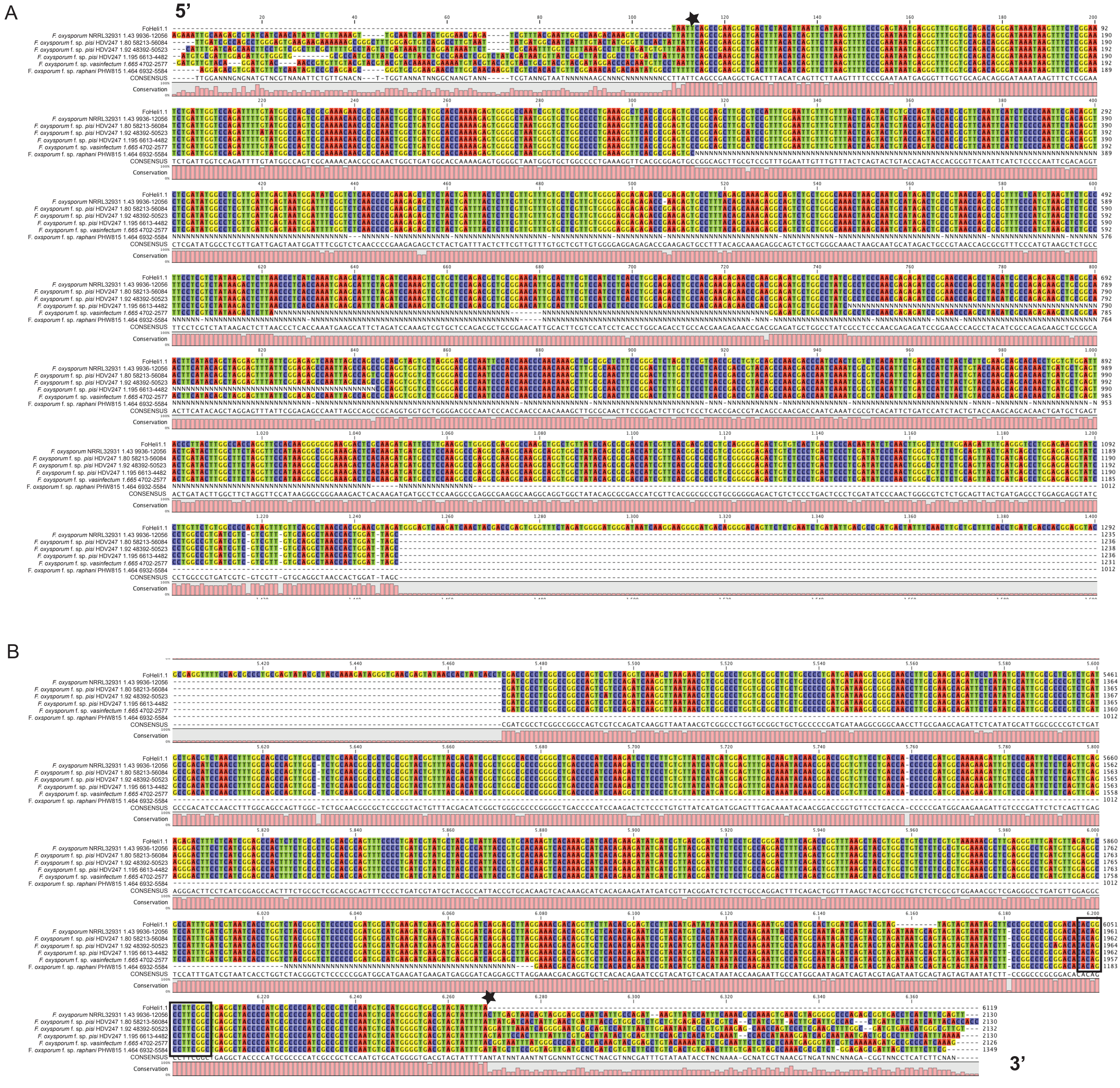

Figure S6: Alignment of non-autonomous element FoHeliNA2 with FoHeli1 shows it derived from FoHeli1.

Multiple sequence alignments of the 5' (A) and 3' termini (B) of 6 FoHeliNA2 sequences (see Table S2 for exact coordinates of these sequences) and FoHeli1.1. Nucleotides are coloured as follows: A red, T green, G yellow, C blue. Start and end of FoHeli(NA)s are indicated with black stars, terminal inverted repeats with grey boxes. The first 1092 and last 837 bp of FoHeliNA2 are ~90% identical to FoHeli1 termini. In contrast to FoHeliNA1, FoHeliNA2 has no additional sequence.

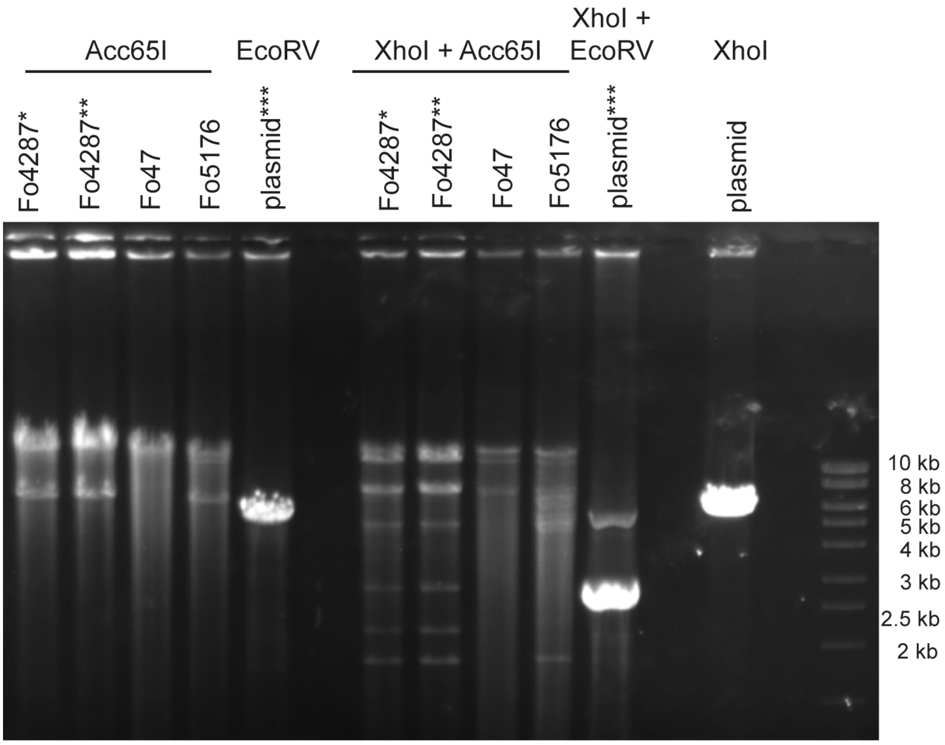

* 250 ng
** 80 ng
***spiked into 80 ng Fo47

Figure S7 Rolling circle amplification (RCA) and digestion with different enzymes.
A. RCA on two different concentrations of genomic DNA (gDNA) of Fol4287 and 80ng of Fo5176 in which we expect that FoHeli is still active, gDNA of one isolate for which we predict that FoHeli1 is not active (Fo47, 80ng) as a negative control and a 5169 bp plasmid spiked into 80 ng of Fo47 gDNA as a positive control. We cut the RCA products with Acc65I, and the plasmid with EcoRV because it does not have the Acc65I restriction site. This resulted in a band of 6-7 kb fragments, which is within the size range we would expect for FoHeli1 and FoHeli2 for the samples for which we expect FoHelis to be active (lanes 1, 2 and 4) and no bands for the negative control (Lane 3). However, double digestion of the RCA products with Acc651 and XhoI results in patterns that cannot be related to FoHeli1 (Lanes 6-10). Next, we cut out the 6-7 kb bands from the Acc651 cut Fol4287 gDNA samples and cloned these. Due to low cloning efficiency only 8 transformants were obtained. The inserts of these were sent out for sequencing. All insert sequences map to distinct regions on the Fol4287 genome and none corresponds to a FoHeli. The insert of one clone maps to mitochondrial DNA. When we then compare the band patterns we obtained with the double digest to what we would expect if we had amplified mitochondrial DNA, we find that these patterns correspond. This suggests the lack of detection of FoHeli fragments may be explained by out-competition by the much more abundant mitochondrial DNA during RCA. To check for the presence of FoHeli sequences in the 6-7 kb fragments, we use an PCR approach with the fragment DNA as template (Figure S8).

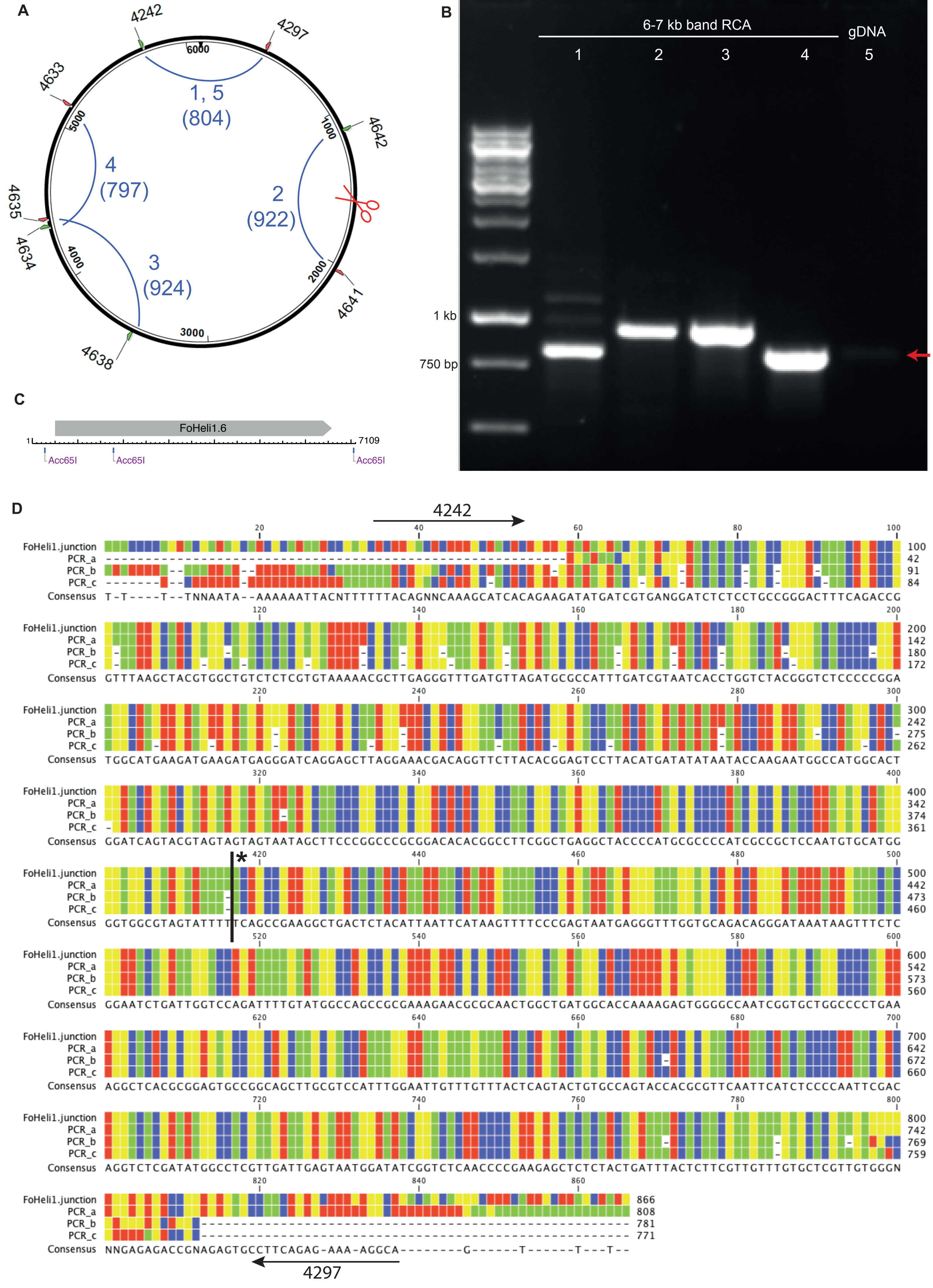
A
4242
4297
6000
1, 5
(804)
4633
5000
4642
1000
4
(797)
2
(922)
4635
4634
4000
3
(924)
2000
4641
3000
4638
B
6-7 kb band RCA
gDNA
1
2
3
4
5
1 kb
750 bp
C
FoHeli1.6
1
7109
Acc65I
Acc65I
Acc65I
D
4242
FoHeli1.junction
PCR_a
PCR_b
PCR_c
Consensus
T-T----T--TNNAATA--AAAAAATTACNTTTTTTACAGNNCAAAGCATCACAGAAGATATGATCGTGANGGATCTCTCCTGCCGGGACTTTCAGACCG
GTTTAAGCTACGTGGCTGTCTCTCGTGTAAAAACGCTTGAGGGTTTGATGTTAGATGCGCCATTTGATCGTAATCACCTGGTCTACGGGTCTCCCCCGGA
TGGCATGAAGATGAAGATGAGGGATCAGGAGCTTAGGAAACGACAGGTTCTTACACGGAGTCCTTACATGATATATAATACCAAGAATGGCCATGGCACT
GGATCAGTACGTAGTAGTAGTAATAGCTTCCCGGCCCGCGGACACACGGCCTTCGGCTGAGGCTACCCCATGCGCCCCATCGCCGCTCCAATGTGCATGG
*
GGTGGCGTAGTATTTTTCAGCCGAAGGCTGACTCTACATTAATTCATAAGTTTTCCCGAGTAATGAGGGTTTGGTGCAGACAGGGATAAATAAGTTTCTC
GGAATCTGATTGGTCCAGATTTTGTATGGCCAGCCGCGAAAGAACGCGCAACTGGCTGATGGCACCAAAAGAGTGGGGCCAATCGGTGCTGGCCCCTGAA
AGGCTCACGCGGAGTGCCGGCAGCTTGCGTCCATTTGGAATTGTTTGTTTACTCAGTACTGTGCCAGTACCACGCGTTCAATTCATCTCCCCAATTCGAC
AGGTCTCGATATGGCCTCGTTGATTGAGTAATGGATATCGGTCTCAACCCCGAAGAGCTCTCTACTGATTTACTCTTCGTTGTTTGTGCTCGTTGTGGGN
NNGAGAGACCGNAGAGTGCCTTCAGAG-AAA-AGGCA--------G------T------T---T--
4297

Figure S8 PCR amplification with FoHeli1-specific primers of 6-7kb RCA fragments.

We used 4 different sets of primers from FoHeli1 to determine whether the 6-7 kb bands we obtained after digestion of RCA products with Acc65I (Figure S7) contain FoHeli1. A. Numbers in blue correspond to lanes in Figure B and numbers in parentheses indicate the expected size of the amplicon. The position of the restriction site of Acc651 is indicated with red scissors. B. The first four lanes represent different primer combinations (see A) on DNA isolated from the cut out band from the RCA experiment in which we digested the DNA with Acc651 (Figure S8). Lanes 1, 3 and 5 give fragments of the expected sizes. In lane 2 we did not expect an amplicon because of the presence of an Acc651 site in between the position of the two primers (A), yet we've found a band whose size corresponds to the length of the sequence between these primers. One way to explain this result is by assuming that the Acc651 digestion of the RCA products (and the gDNA) was not complete which might have resulted in a 6-7 kb gDNA fragment containing FoHeli1.6. In the genome this copy of FoHeli1 is flanked by two Acc651 sites (C). Hence, the fragment in lane 2 may originate from amplification of non-digested FoHeli1.6 DNA .D. The fragment of lane 1 should contain joined ends originally derived from a circular FoHeli DNA form. To check this, the fragment was sequenced. Alignment of the PCR product sequence with FoHeli1 indicates the presence of joined ends indeed. The junction is indicated with a black line and arrow. The fifth lane of B shows the amplicon for primer combination 4242+4297, but the band is hardly visible. This amplicon corresponds to FoHeli1 joined ends, on Fo4287 genomic DNA without RCA, thus repeating part of the PCR experiment from Figure 4 in the main manuscript (red arrow).

Primer sequences:

Number	Sequence
4297	TGCCTCTTTGCTCTGAAGG
4242	ACAAGTCACAAAGCATCAC
4642	TTCAGGCTAACCACGGAACG
4641	TTGCCGACTAAGAATTGC
4633	TGCGAAACGCCTCCTGGTC
4634	ACTGGAGGAAGAGGACATCG
4635	TCCAATCGTAATTGATGTC
4638	AAGGCAGGTCGTACTATGTC

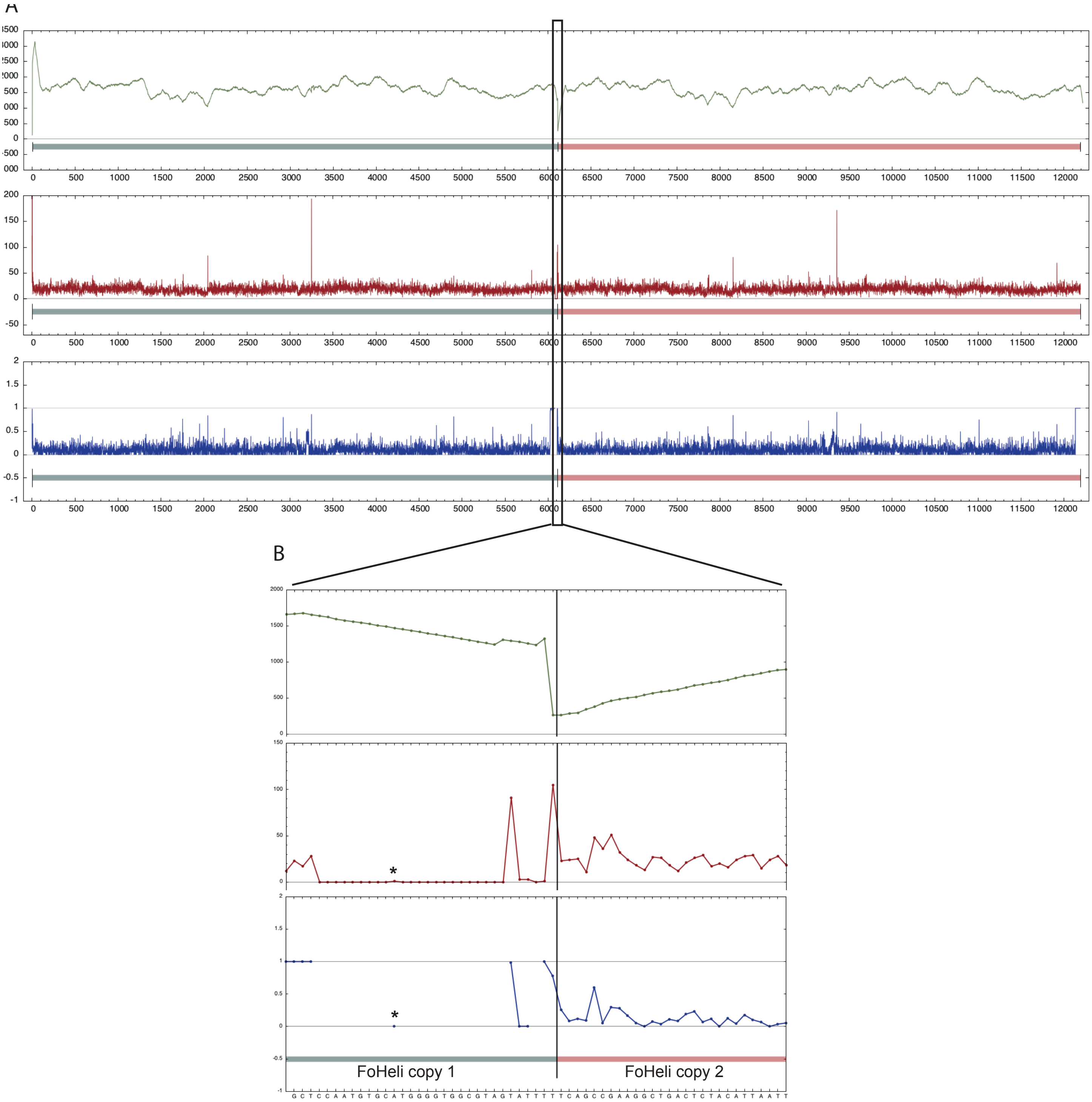

Figure S9 The ‘closed end’ sequence of FoHeli does not occur in the genome of Fol4287: Illumina sequencing reads mapped as single-end reads.

To exclude the possibility that the sequences from Figure 3C and Figure S9 stem from a tandem insertion of FoHeli1 rather than an excised FoHeli1 with joined ends, we map 230,515 out of 4,383,674 reads from 3 different Illumina sequencing libraries on a constructed sequence of two FoHeli1 in tandem. We added ‘TNAT’ to the 5’ end of the first copy to include part of the FoHeli1 the insertion site. To achieve maximum detection sensitivity we map the reads as single-end and allow for partial mapping (clipping) of reads. We used three different libraries of paired reads obtained selecting for different insert sizes (i.e. distance between two paired reads: 170, 500 and 5000 bp). To map them as single end we simply concatenated the 6 fastq files (two for each library, each containing one half of a read pair) and mapped these reads on our constructed tandem insertion sequence using **bwa mem** with default settings (Li and Durbin 2009). A. Read density per position (top panel, green), number of mapping starts (= leftmost position of read mapping) per position and the fraction of mapping starts that correspond to mapped reads that are soft or hard clipped (hence partially mapped). The average read density is ~1500 on the two copies, given a genome-wide average read density of ~100, we estimate that Fol4287 has 30 full-length FoHeli1 copies, 6 more than our lower-bound estimate based on 5’ partial sequences (Table S4) B. Same as in A, but zoomed in on the junction between tandem FoHeli1s. The reads density drops steeply at this junction. We expect a steep drop if a tandem insertion occurs only once and ‘stand alone’ insertion occurs ~28 times. However, the two panels below show that no mappings start in the 3’ region of the leftmost FoHeli, and that those that do are all clipped (i.e. partially mapped: part of the sequence does not match the reference) . Only a single read (indicated with a *) spans the junction more than 6 bp and is completely mapped (with one deletion and one mutation).

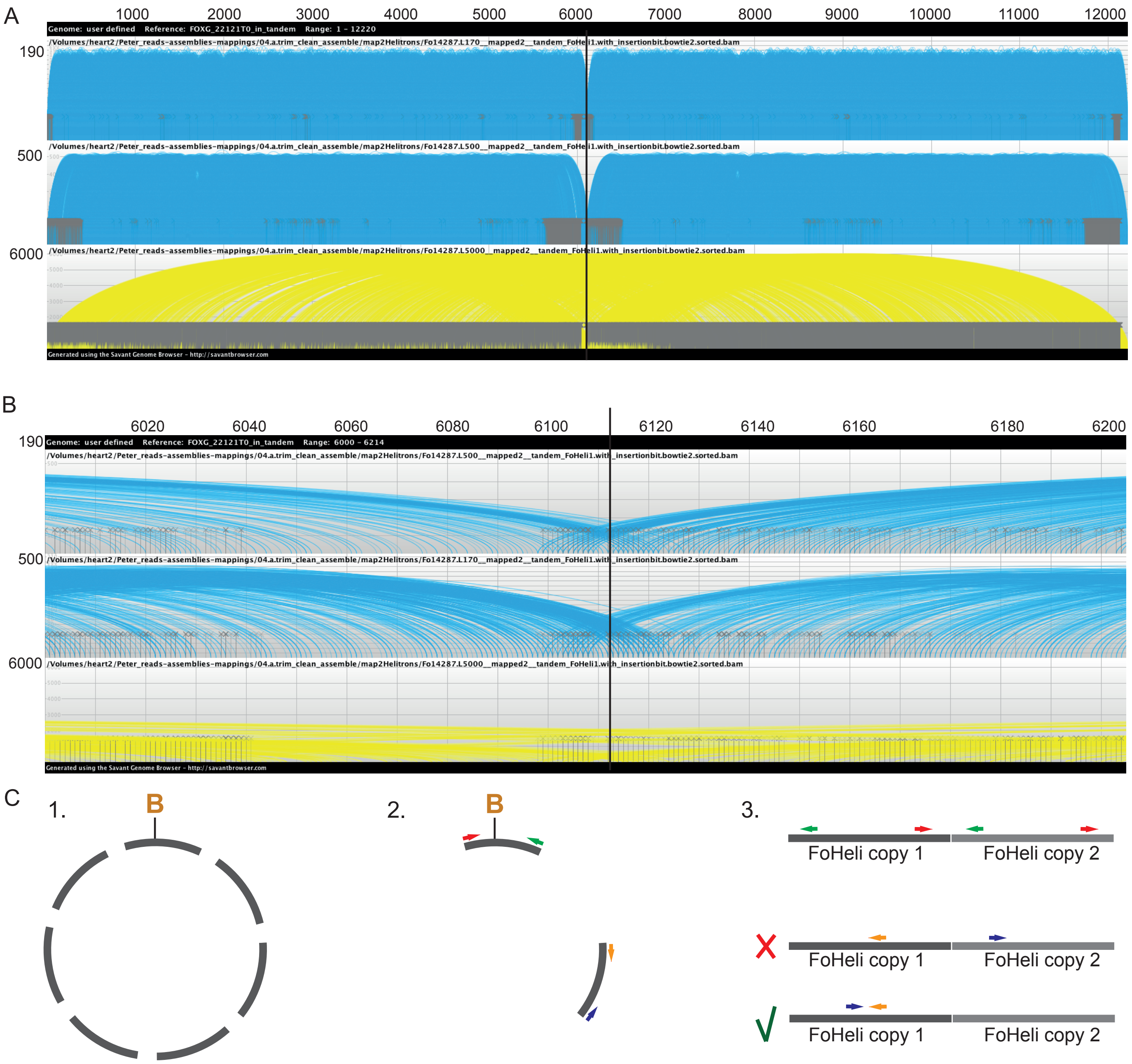

Figure S10 The 'closed end' sequence of FoHeli does not occur in the genome of Fol4287: Illumina sequencing reads mapped as paired reads.

If FoHeli occurs as a tandem insertion in Fol4287, we expect that if we map paired reads on a constructed sequence of FoHeli1 in tandem some read pairs will span the junction of the two copies even if the junction sequence itself is not present in any of the libraries (Figure S10) . We use bowtie2 (Langmead et al. 2009) to map reads from the same three Illumina sequencing libraries mentioned above onto the same FoHeli1 tandem constructed sequence as we used above (Figure S10), using the following settings: 170 bp insert size: min. insert size 140, max. insert size 200, forward-reverse orientation (paired-end), 500 bp insert size: min. insert size 400, max. insert size 600, forward-reverse orientation (paired-end), 5000 bp insert size: min. insert size 4000, max. insert size 6000, reverse-forward orientation (mate-pair). We use the Savant genome browser (Fiume et al. 2012) to depict read pairs as arcs and look for arcs that bridge the junction between the two FoHeli1 copies. A. Arc view of mapped reads on the complete FoHeli1 tandem constructed sequence. The top panel depicts reads from the library with insert size 170 (paired-end:blue), the middle panel depicts reads from the library with insert size 500 (paired-end:blue) and the bottom panel reads from the library with insert of ~5 kb (mate-pair: yellow). Reads for which the mate was not mapped are depicted in grey. The black vertical bar indicates the position of the junction of the two FoHeli copies. B. Same as Figure A but zoomed in on the junction sequence. We find that for the libraries with the smallest insert sizes no read pair bridges the junction between the two FoHeli1 copies. In contrast, for the large insert-size library we find 1314 read pairs that bridge the junction, but this may also be explained by contamination of the large insert mate-pair library with short insert paired-end reads that represent non-biotinylated fragments that were not properly removed during the wash step in library preparation (see C). C. To generate mate-pair libraries: (1) 5 kb fragments are circularized using biotin and sheared, (2) the sample is washed to remove non-biotinylated fragments, but some non-biotynilated fragments may remain in the sample (Sahlin et al. 2016). Primers anneal to remaining fragments. (3) Paired reads are mapped to the tandem FoHeli sequence where a read-pair points outwards and reads are spaced ~5 kb apart. These settings cause paired-end contamination to be mapped across two FoHeli1 copies, which explains why we don't find reads from the two small insert size libraries that map across the junction. Correct mate-pairs that for which both reads map within a FoHeli sequence can be mapped in two locations (one per copy, top panel), in this case one location is chosen randomly.

A. Multiple 5' termini of FoHeli1 in *F. oxysporum* f. sp. *conglutinans* PHW808

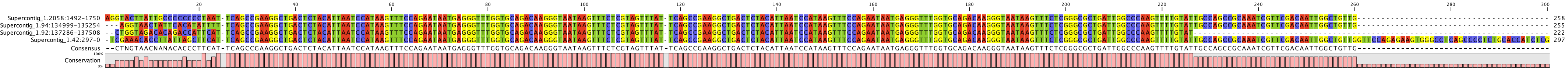

B. Multiple 5' termini of FoHeli2 in *F. oxysporum* f. sp. *conglutinans* PHW808

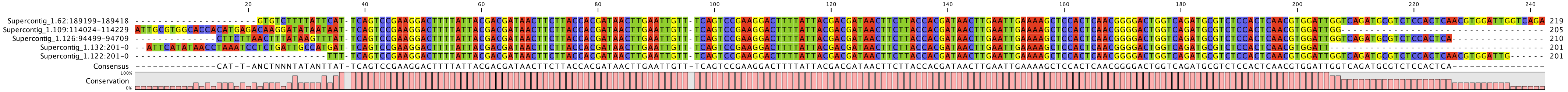

C. Multiple 5' termini of FoHeli4 in *F. oxysporum* f. sp. *conglutinans* PHW808

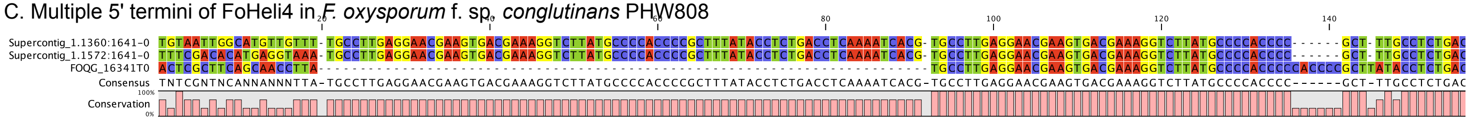

Figure S11: Helitrons with multiple 5' termini have transposed.
A-C. Multiple sequence alignment of partial FoHelis with some 5' flanking sequence demonstrates that different copies of these chaemeric Helitrons arose thorugh transposition, not segmental duplication, as sequence similarity drops at the end of the Helitron elements. We selected sequences from *Fusarium oxysporum* f. sp. *conglutinans* PHW808, but this holds true for all Helitrons with multiple 5' termini we have identified in this study (see Table S6 for a full list).
D. Cartoon on how Helitrons with multiple termini could arise.

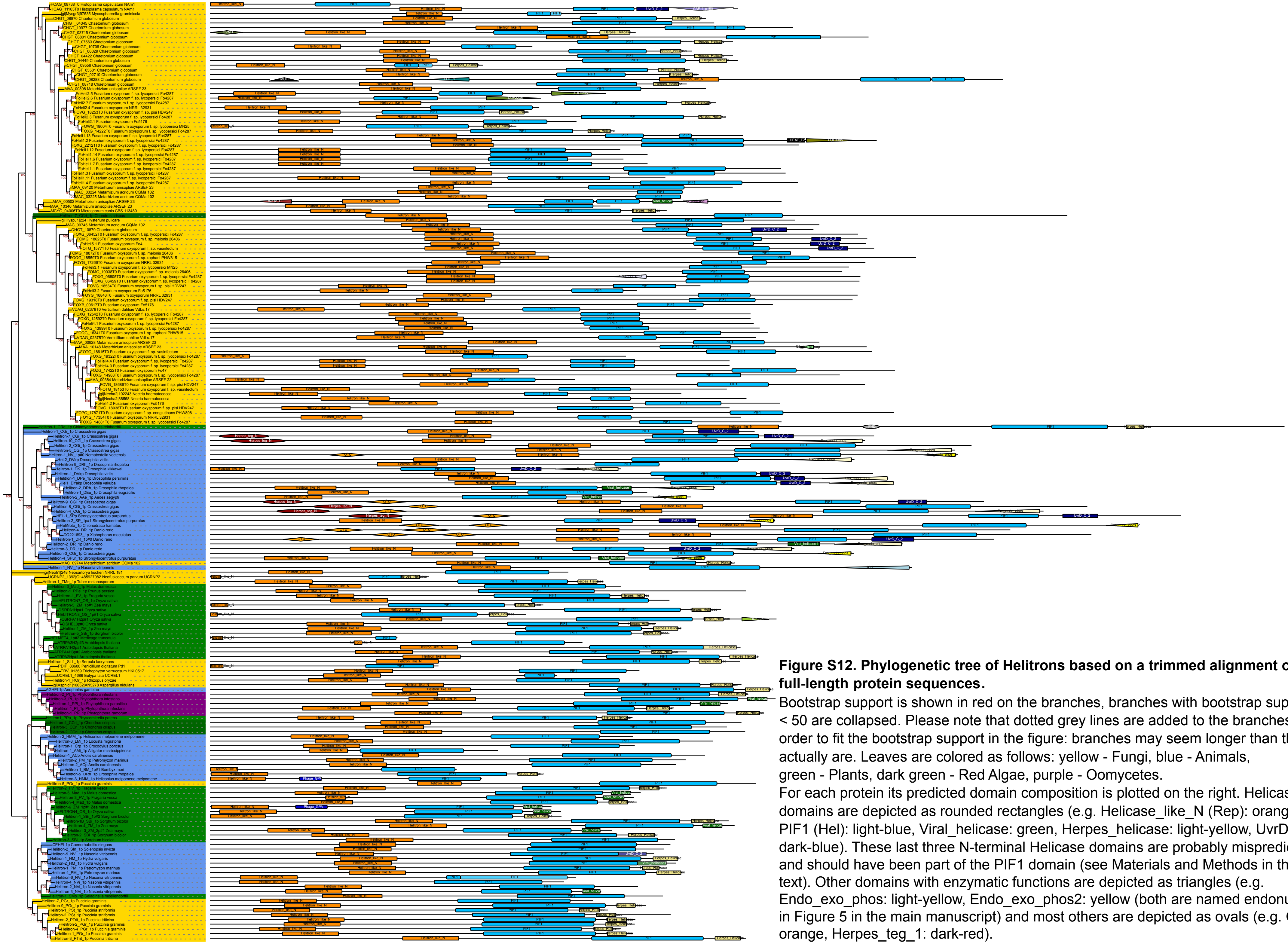

Figure S12. Phylogenetic tree of Helitrons based on a trimmed alignment of full-length protein sequences.

Bootstrap support is shown in red on the branches, branches with bootstrap support < 50 are collapsed. Please note that dotted grey lines are added to the branches in order to fit the bootstrap support in the figure: branches may seem longer than they actually are. Leaves are colored as follows: yellow - Fungi, blue - Animals, green - Plants, dark green - Red Algae, purple - Oomycetes.
For each protein its predicted domain composition is plotted on the right. Helicase-like domains are depicted as rounded rectangles (e.g. Helicase_like_N (Rep): orange, PIF1 (Hel): light-blue, Viral_helicase: green, Herpes_helicase: light-yellow, UvrD_C_2: dark-blue). These last three N-terminal Helicase domains are probably mispredictions, and should have been part of the PIF1 domain (see Materials and Methods in the main text). Other domains with enzymatic functions are depicted as triangles (e.g. Endo_exo_phos: light-yellow, Endo_exo_phos2: yellow (both are named endonuclease in Figure 5 in the main manuscript) and most others are depicted as ovals (e.g. OTU: orange, Herpes_teg_1: dark-red).

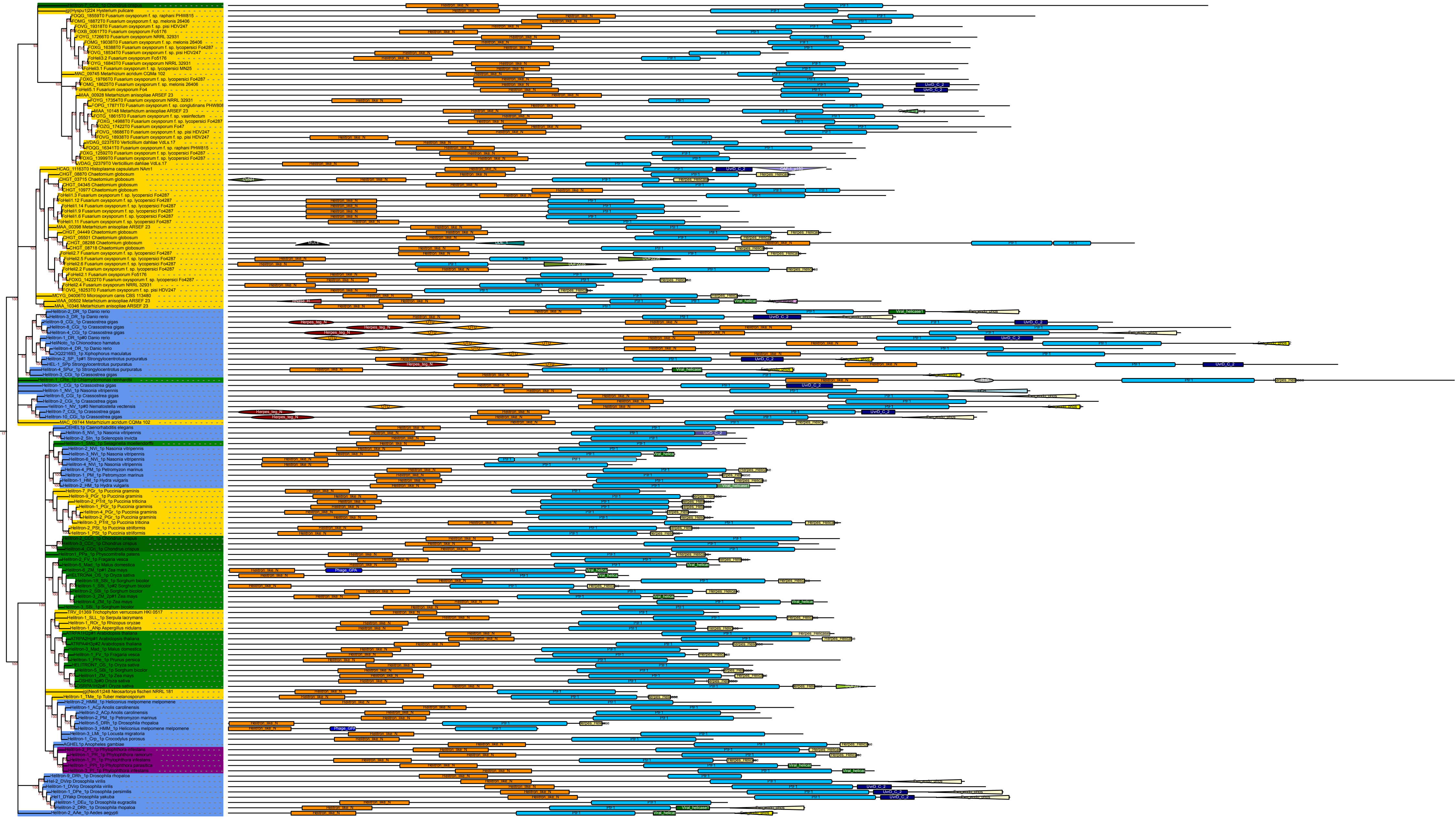

Figure S13 Phylogenetic tree of Helitron proteins based on alignment of the Rep and Hel domain.

Phylogenetic tree based on alignment by hmmalign of predicted Rep and Hel domains. Bootstrap support is shown in red on the branches. Please note that dotted lines are added in order to fit bootstrap support in the figure, dotted lines are added, this means that some branches appear to be longer than they are. Branches with bootstrap support < 50 are collapsed.

Leaves are colored as follows: yellow - Fungi, blue - Animals, green - Plants, dark green - Red Algae, purple - Oomycetes. For each protein its predicted domain composition is on the right. Helicase-like domains are depicted as rounded rectangles (e.g. Helicase_like_N: orange, PIF1: light-blue, Viral_helicase: green, Herpes_helicase: light-yellow, UvrD_C_2: dark-blue). These last three N-terminal Helicase domains are probably mispredictions, and should have been part of the PIF1 domain (see Materials and Methods in the main text). Other domains with enzymatic functions are depicted as triangles (e.g. Endo_exo_phos: light-yellow, Endo_exo_phos2: yellow (both are named endonuclease in Figure 5 in the main manuscript) and most others are depicted as ovals (e.g. OTU:orange, Herpes_teg_1: dark-red).

In this tree, Helentrons are not monophyletic.

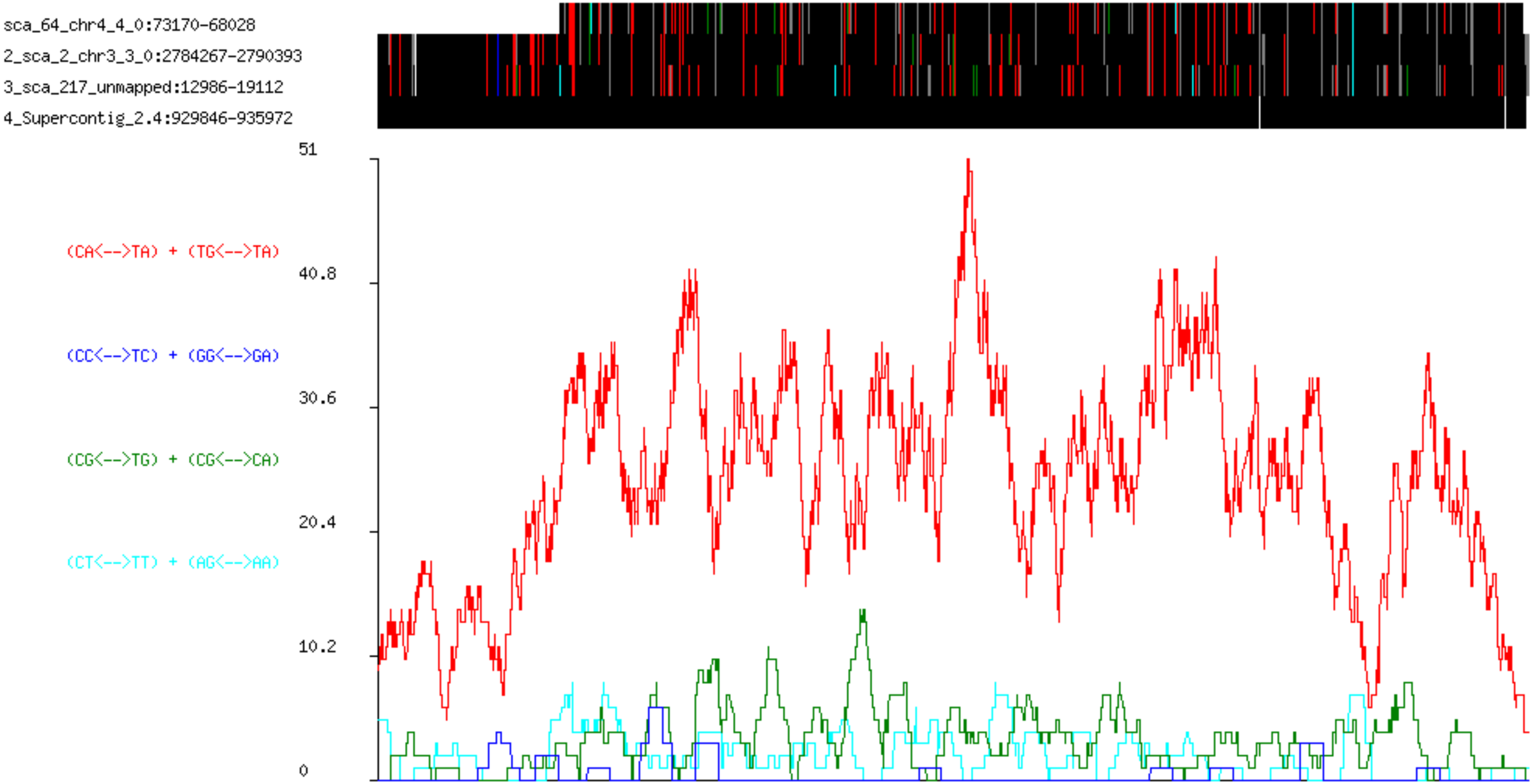

Figure S14 Putative RIP mutations in *F. solani* FoHeli1 homologs.
We aligned three putative FoHeli1 homologs in *F. solani* (the top three sequences in the top panel) and FoHeli1.12 (bottom sequence in top panel) using clustalo. Each type of RIP-associated CpN <-> TpN mutation has its own color: CpA <-> TpA in red, CpC <->TpC in blue, CpG <-> TpG in green and CpT <-> TpT in cyan. In the top panel, mutations in F. solani FoHeli1 homologs are coloured according to the type of mutation and most are red. The bottom panel shows the number of RIP-associated mutations over a 50 bp scanning window. Most RIP-associated changes in the three *F. solani* sequences, are CpA to TpA and TpG to TpA.

References

Delcher AL, Phillippy A, Carlton J, Salzberg SL. 2002. Fast algorithms for large-scale genome alignment and comparison. Nucleic Acids Res. 30:2478-2483.

Fairman-Williams ME, Guenther UP, Jankowsky E. 2010. SF1 and SF2 helicases: family matters. Curr Opin Struct Biol. 20:313-324.

Fiume M, Smith EJ, Brook A, Strbenac D, Turner B, Mezlini AM, Robinson MD, Wodak SJ, Brudno M. 2012. Savant Genome Browser 2: visualization and analysis for population-scale genomics. Nucleic Acids Res. 40:W615-21.

Langmead B, Trapnell C, Pop M, Salzberg SL. 2009. Ultrafast and memory-efficient alignment of short DNA sequences to the human genome. Genome Biol. 10:R25-2009-10-3-r25. Epub 2009 Mar 4.

Li H, Durbin R. 2009. Fast and accurate short read alignment with Burrows-Wheeler transform. Bioinformatics. 25:1754-1760.

Koonin EV, Corbalenya AE. 1993. Helicases: amino acid sequence comparisons and structure-function relationships. Current Opinion in Structural Biology. 3:419-429.

Koonin EV, Ilyina TV. 1993. Computer-assisted dissection of rolling circle DNA replication. Biosystems. 30:241-268.

Ma LJ, van der Does HC, Borkovich KA, Coleman JJ, Daboussi MJ, Di Pietro A, Dufresne M, Freitag M, Grabherr M, Henrissat B et al. 2010. Comparative genomics reveals mobile pathogenicity chromosomes in Fusarium. Nature. 464:367-373.

Sahlin K, Chikhi R, Arvestad L. 2016. Assembly scaffolding with PE-contaminated mate-pair libraries. Bioinformatics
